# Targeting glutamine metabolism slows soft tissue sarcoma growth

**DOI:** 10.1038/s41467-020-14374-1

**Published:** 2020-01-24

**Authors:** Pearl Lee, Dania Malik, Nicholas Perkons, Peiwei Huangyang, Sanika Khare, Seth Rhoades, Yao-Yu Gong, Michelle Burrows, Jennifer M. Finan, Itzhak Nissim, Terence P. F. Gade, Aalim M. Weljie, M. Celeste Simon

**Affiliations:** 1 Abramson Family Cancer Research Institute, University of Pennsylvania Perelman School of Medicine, Philadelphia, PA 19104 USA; 2 Department of Systems Pharmacology and Translational Therapeutics, University of Pennsylvania, Philadelphia, PA 19104 USA; 3 Institute of Translational Medicine and Therapeutics, Perelman School of Medicine, University of Pennsylvania, Philadelphia, PA 19104 USA; 4 Department of Bioengineering, University of Pennsylvania, Philadelphia, PA 19104 USA; 5 Division of Genetics and Metabolism, Children’s Hospital of Philadelphia, Philadelphia, PA 19104 USA; 6 Department of Pediatrics, Biochemistry, and Biophysics, University of Pennsylvania, Philadelphia, PA 19104 USA; 7 Department of Radiology, University of Pennsylvania Perelman School of Medicine, Philadelphia, PA 19104 USA; 8 Department of Cell and Developmental Biology, University of Pennsylvania Perelman School of Medicine, Philadelphia, PA 19104 USA

**Keywords:** Cancer, Cancer metabolism, Sarcoma, Cancer metabolism

## Abstract

Tumour cells frequently utilize glutamine to meet bioenergetic and biosynthetic demands of rapid cell growth. However, glutamine dependence can be highly variable between in vitro and in vivo settings, based on surrounding microenvironments and complex adaptive responses to glutamine deprivation. Soft tissue sarcomas (STSs) are mesenchymal tumours where cytotoxic chemotherapy remains the primary approach for metastatic or unresectable disease. Therefore, it is critical to identify alternate therapies to improve patient outcomes. Using autochthonous STS murine models and unbiased metabolomics, we demonstrate that glutamine metabolism supports sarcomagenesis. STS subtypes expressing elevated glutaminase (GLS) levels are highly sensitive to glutamine starvation. In contrast to previous studies, treatment of autochthonous tumour-bearing animals with Telaglenastat (CB-839), an orally bioavailable GLS inhibitor, successfully inhibits undifferentiated pleomorphic sarcoma (UPS) tumour growth. We reveal glutamine metabolism as critical for sarcomagenesis, with CB-839 exhibiting potent therapeutic potential.

## Abstract (web summary)

Glutamine is an energetic source required for the proliferation of cancer cells. Here, the authors show that soft tissue sarcomas expressing high levels of glutaminase (GLS) are particularly sensitive to glutamine starvation and GLS inhibition in tumour-bearing allograft and autochthonous mouse models.

## Introduction

Soft tissue sarcomas (STSs) are a diverse group of malignancies arising from mesenchymal tissues, now classified into >80 different subtypes based on their tissue of origin, genetic alterations, and age of occurrence^1,2^. The annual incidence of STS (13,000 new cases) is comparable to oesophageal (17,500 new cases) and cervical (12,000 new cases) cancers^3,4^, and accounts for a disproportionate share of cancer mortality among young adults^5^. The three most common STSs detected in the extremities and retroperitoneum are liposarcomas (20–25%), leiomyosarcomas (14%), and undifferentiated pleomorphic sarcomas (UPS; 14%)^4,6–8^. For localized cases with 81% five-year survival rates, surgical resection, usually accompanied by radiation and chemotherapy, remains the primary treatment option. In contrast, cytotoxic chemotherapy typically represents the therapeutic approach for metastatic or unresectable STS, where 16% five-year survival rates are observed^9–11^. While substantial progress has revealed genetic features of distinct sarcoma subtypes^2,5,12^, these diseases remain relatively understudied, making improved therapeutics necessary and challenging.

Cancer cells typically rewire their metabolism to meet the bioenergetic and biosynthetic demands of uncontrolled cell growth^13–15^. For example, many oncogenic mutations result in enhanced glucose metabolism, reflecting its importance to tumour cell proliferation. Glutamine, the most abundant circulating amino acid, is also highly metabolized by many cancer cells for various purposes^16,17^, including the generation of tricarboxylic acid (TCA) cycle intermediates and amino acids, and maintaining redox homoeostasis^18–22^. Therefore, targeting glutamine metabolism is an appealing therapeutic option in a number of cancer subtypes^17^.

Glutaminase (GLS), the enzyme converting glutamine to glutamate, primarily enhances glutaminolysis^22–24^. Compounds disrupting glutamine metabolism currently exist, such as the allosteric GLS inhibitor BPTES^25,26^, which has been studied in lymphoma, liver, and renal cancers^27–29^. However, BPTES’ moderate potency and low bioavailability limit clinical potential.

In contrast, Telaglenastat (CB-839) represents a potent, orally bioavailable GLS inhibitor showing anti-tumour activity in transplantable cancer models, including breast cancer and lymphoma^30,31^, and ongoing clinical trials are examining efficacy in haematologic and solid cancers^32^. Nevertheless, caution is warranted, given that effectiveness of GLS inhibition appears to be cancer-type specific. Mutant *Kras* and *Trp53*-deleted (*LSL-Kras*^*G12D/+*^;*Trp53*^*fl/fl*^; *KP*) lung adenocarcinomas do not exhibit preferential glutamine metabolism in vivo. Instead, glucose-derived pyruvate is important, despite glucose oxidation being dispensable in vitro^33^. In pancreatic ductal adenocarcinoma (PDAC) models, similarly exhibiting *Kras*^*G12D*^ and altered p53 status (*Trp53*^*fl/+*^), multiple adaptive responses support GLS-independent cell growth^34^. Collectively, tumour microenvironment, surrounding tissue, and cell of origin influence metabolic responses in these models that mitigate the effects of CB-839.

Here, we employ a UPS mouse model harbouring mutations identical to previously characterized lung and PDAC animals. *KP* mice generate temporally and spatially restricted hindlimb tumours that metastasize to the lung and accurately mimic human disease on histological, transcriptional, and pathological levels^35–38^. Furthermore, we overlay HIF-2α loss to generate *LSL-Kras*^*G12D/+*^;*Trp53*^*fl/fl*^*;Epas1*^*fl/fl*^ (*KPH2*) animals^38^, where HIF-2α surprisingly suppresses sarcomagenesis^38^. Animals deficient in aryl hydrocarbon receptor nuclear translocator (ARNT), the common HIF-1α and HIF-2α binding partner, are also used to generate *LSL-Kras*^*G12D/+*^;*Trp53*^*fl/fl*^*;Arnt*^*fl/fl*^ (*KPA*) cohorts. All three models faithfully recapitulate the human disease, allowing for comparisons between healthy muscle, primary UPS *KP* tumours, larger *KPH2* sarcomas, and even bigger *KPA* tumours. *KP*, *KPH2*, and *KPA* samples were subjected to unbiased metabolomic screens to analyse metabolic pathways promoting sarcomagenesis based on overall tumour size. We determine that glutamine metabolism intermediates are strikingly elevated in *KPH2* and *KPA* tumours compared to normal muscle, and STS cell line growth is compromised under glutamine deprivation. Notably, STSs expressing high GLS exhibit increased dependency on glutamine, required to support the TCA cycle, aspartate production, and subsequently, nucleotide synthesis for tumour cell growth. GLS inhibition with CB-839 successfully targets GLS-expressing cells. Based on previous studies where CB-839 effects were not recapitulated in vivo, we anticipated minor effects on *KP* sarcomas. However, CB-839 significantly reduces tumour growth in various UPS models in vivo. These aligning in vitro and in vivo results are in stark contrast to previous lung and PDAC models, suggesting that cell of origin is more important to the tumour metabolic millieu than driver mutations (i.e. *KRAS* and *TP53*). Altogether, the virtual lack of targeted STS therapies makes GLS inhibitors highly promising, particularly in STS patients stratified by their GLS expression, maximizing potential therapeutic efficacy of GLS inhibition.

## Results

### UPS and other STS subtypes show glutamine dependency

**Fig. 1:**
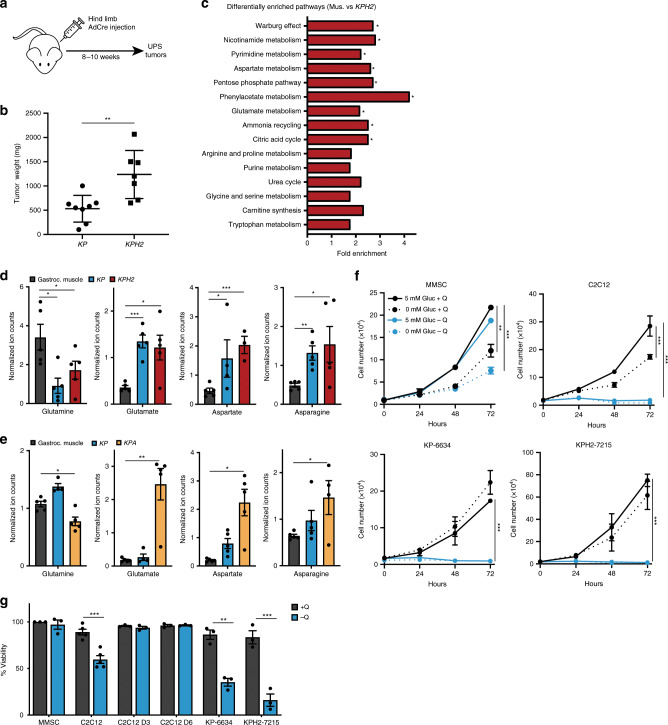
UPS tumours and cells exhibit evidence of glutamine dependency. **a** Undifferentiated pleomorphic sarcoma (UPS) tumours are generated by injection of adenovirus expressing Cre-recombinase (AdCre) into hindlimb muscles of *LSL-Kras*^*G12D/+*^*;Trp53*^*fl/fl*^ (*KP*) and *LSL-Kras*^*G12D/+*^*;Trp53*^*fl/fl*^*;Epas1*^*fl/fl*^ (*KPH2*) mice, and harvested 8–10 weeks after viral delivery. **b** Tumour weights of *KP* (*n* = 8) and *KPH2* tumours (*n* = 7) at 9 weeks post-AdCre injection. Error bars are ± s.d; **p < 0.005. **c** MetaboAnalyst pathway enrichment analysis of VIP > 1 metabolites in muscle (Mus.) and *KPH2* comparison following LC/MS; *p < 0.05. *N* = 4 with three technical replicates. **d** Normalized ion counts for glutamine, glutamate, aspartate, and asparagine (left to right) in gastrocnemius muscle (gastroc. muscle), *KP*, and *KPH2* tumours. *N* = 4 with three technical replicates. Data represent mean ± s.d; *p < 0.05, **p < 0.005, ***p < 0.0005. **e** Normalized ion counts for glutamine, glutamate, aspartate, and asparagine (left to right) in gastroc. muscle, *KP*, and *LSL-Kras*^*G12D/+*^*;Trp53*^*fl/fl*^*;Arnt*^*fl/fl*^ (*KPA)* tumours. *N* = 4 with three technical replicates; *p < 0.05, **p < 0.005. **f** Proliferation of murine mesenchymal stem cells (MMSC; top left), murine myoblasts (C2C12; top right), *KP* tumour-derived cells (KP-6634; bottom left), and *KPH2* tumour-derived cells (KPH2-7215; bottom right) grown in media with or without glucose (Gluc) and/or glutamine (Q). *N* = 3 with three technical replicates each. Data represent mean ± s.e.m.; **p < 0.005, ***p < 0.0005. **g** Viability of MMSCs, C2C12s, differentiated C2C12s (3 days, C2C12 D3; 6 days, C2C12 D6), KP-6634s, and KPH2-7215s grown in media with or without Q and assessed after 48 h. *N* = 3 with two technical replicates. Data represent mean ± s.e.m; **p < 0.005, ***p < 0.0005. *p*-Values were calculated from a two-tailed Student’s *t*-test for **b**, **d**–**g**. Source data are provided as a Source Data file.

To study metabolic differences between skeletal muscle and UPS tumours, an autochthonous *LSL-Kras*^*G12D/+*^;*Trp53*^*fl/fl*^ (*KP)* UPS mouse model was utilized. Injection of adenovirus expressing Cre-recombinase (AdCre) into hindlimb musculature induces mutant *Kras* expression, *Trp53* loss, and development of UPS tumours (Fig. 1a)^35–38^. We previously expanded upon this model with additional HIF-2α loss to generate *LSL-Kras*^*G12D/+*^;*Trp53*^*fl/fl*^*;Epas1*^*fl/fl*^ (*KPH2*) animals, and determined HIF-2α surprisingly suppresses tumourigenesis^38^ (Fig. 1b). Moreover, decreased *HIF-2α* mRNA expression was detected in a majority of STS patient samples compared to normal adipose tissue, suggesting that *EPAS1* is epigenetically silenced^38^. As *KP* and *KPH2* models faithfully recapitulate human disease and rapidly form spatially controlled tumours, both were utilized for the purpose of dissecting distinct metabolic pathways enhancing UPS growth. While *KPH2* tumours are most representative of human STSs and significantly larger than *KP* tumours, examining metabolic changes in *KP* samples provides another level of insight into metabolic changes that may occur during earlier stages of sarcomagenesis.

Pan-metabolomic analysis was performed on gastrocnemius muscle, *KP*, and *KPH2* sarcomas. We employed principal component analysis (PCA) to identify metabolic alterations between muscle (WT; green), *KP* (blue), and *KPH2* (red) tumours, and each cohort separated into relatively distinct clusters (Supplementary Fig. 1A, Supplementary Data 1). Subsequently, orthogonal projections to latent structures discriminant analysis (OPLS-DA) defined metabolites contributing to the greatest separation between groups (Supplementary Fig. 1B). Both *KP* and *KPH2* tumours had markedly distinct metabolic profiles compared to muscle; while also separating from one another in the OPLS-DA model, although this was not statistically significant. Metabolites distinguishing gastrocnemius muscle (Mus.) and *KPH2* tumours (VIP > 1) were assessed (Supplementary Fig. 1B), and those involved in amino acid metabolism, nucleotide synthesis, and the pentose phosphate pathway largely contributed to their separation (Fig. 1c, Supplementary Data 2). Moreover, decreased glutamine along with increased glutamate, aspartate, and asparagine abundance was observed in *KP* and *KPH2* tumours, suggesting that glutamine-related metabolism was highly active (Fig. 1d). Despite this observation, levels in essential and other non-essential amino acids, such as arginine and alanine, showed fewer changes between muscle, *KP*, and *KPH2* tumours (Supplementary Fig. 1C, D).

A similar comparison was performed between gastrocnemius muscle, *KP*, and *LSL-Kras*^*G12D/+*^;*Trp53*^*fl/fl*^*;Arnt*^*fl/fl*^ (*KPA*) sarcomas. In the *KPA* model, injection of AdCre in the hindlimb also deletes ARNT, the common binding partner for both HIF-1α and HIF-2α. Resulting *KPA* tumours have even greater primary tumour growth and weight compared to *KPH2* tumours, emphasizing the importance of HIF-1α and HIF-2α loss^38^. PCA and OPLS-DA revealed a clear separation of cohorts between the gastrocnemius muscle (WT; yellow), *KP* (purple), and *KPA* (red) tumours (Supplementary Fig. 1E, F, Supplementary Data 3). Much like *KPH2* analyses, decreased glutamine and increased glutamate, aspartate, and asparagine levels were observed in *KPA* tumours compared to the muscle (Fig. 1e). While essential amino acids showed few differences (Supplementary Fig. 1G), other non-essential amino acids were elevated in *KP* and *KPA* tumours, such as proline and serine (Supplementary Fig. 1H). Analysis with *KP* and *KPA* tumours also revealed a stepwise increase in a number of metabolites (e.g. glutamate and aspartate; Fig. 1e), showing increased growth between genotypes with *KPA* tumours being largest, followed by *KP* tumours, compared to gastrocnemius muscle.

UPS cell glutamine dependency was evaluated in vitro using *KP* and *KPH2* tumour-derived cell lines (KP-6634 and KPH2-7215, respectively) to determine whether metabolic changes observed in vivo were recapitulated in vitro. KPH2-7215 cells proliferate more rapidly than KP-6634s, in line with in vivo growth rates. In addition, two cell types previously implicated as UPS cells of origin: murine mesenchymal stem cells (MMSCs) and murine myoblasts (C2C12) were also examined. Cells were cultured in glucose-free and/or glutamine-free conditions to discern whether a specific nutrient was favoured. MMSCs showed decreased proliferation upon glucose withdrawal, but were largely unaffected by glutamine deprivation unless glucose was simultaneously removed (Fig. 1f). In contrast, C2C12s, KP-6634s, and KPH2-7215s exhibited a critical dependency on glutamine with a decrease in both proliferation and cell viability (Fig. 1f, g). Interestingly, C2C12s are also glucose dependent, as its withdrawal significantly inhibited cell proliferation, unlike KP-6634s and KPH2-7215s. This implies a more normal state for C2C12s, where both glucose and glutamine play a large role in their metabolism. Notably, upon differentiation into myotubes, C2C12s became resistant to glutamine withdrawal (Fig. 1g), suggesting this phenotype is limited to actively proliferating mesenchymal/sarcoma cells.

To test whether these findings extended to human sarcoma cell lines, HT1080 (fibrosarcoma), SK-LMS-1 (leiomyosarcoma), SK-UT-1B (leiomyosarcoma), CCL-136 (rhabdomyosarcoma), LPS246 (dedifferentiated liposarcoma), SW872 (pleomorphic liposarcoma), and T778 (well-differentiated liposarcoma) were similarly cultured in conditions of glucose and/or glutamine deficiency. Whereas HT1080s proliferated more slowly in the absence of glucose, a greater decrease in proliferation and viability was observed upon glutamine deprivation in SK-LMS-1s, SK-UT-1Bs, and CCL-136s (Supplementary Fig. 1I, J). In contrast, while glucose withdrawal affected the proliferation of LPS246s, SW872s were unaffected by either metabolite, although proliferation decreased when both were removed. Unlike the other two liposarcoma cell lines tested, T778s also showed glutamine dependency; however, only cell proliferation was affected, suggesting growth arrest rather than cell death as a consequence of glutamine withdrawal (Supplementary Fig. 1I, J). Interestingly, glutamine dependency was not solely a consequence of increased proliferation rates. HT1080s and SW872s proliferated at similar rates with differential responses to glutamine deprivation. These results demonstrate that glutamine deprivation inhibits in vitro growth and viability of multiple STS cell types, including UPS, fibrosarcoma, and leiomyosarcoma, but not all, particularly liposarcoma sub-types. The significance of these differences in glutamine metabolism will be discussed below.

### UPS cells maintain glutamine anaplerosis

**Fig. 2:**
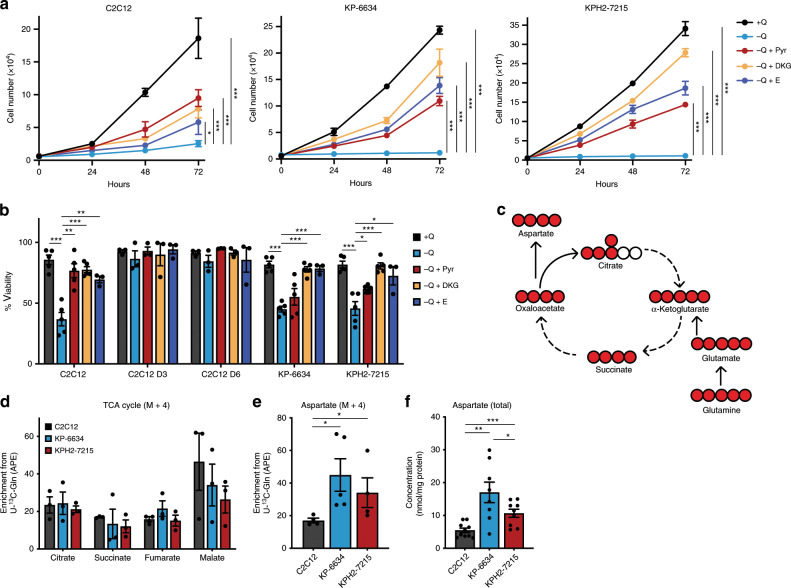
UPS tumours cells maintain glutamine anaplerosis. **a** Proliferation of C2C12s, KP-6634s, and KPH2-7215s (left to right) grown in media with or without glutamine (Q), supplemented with 5 mM pyruvate (Pyr), 5 mM dimethyl 2-oxoglutarate (DKG), or 2 mM glutamate (E). *N* = 3 with three technical replicates. Data represent mean ± s.e.m.; *p < 0.05, ***p < 0.0005. **b** Viability of C2C12, C2C12 D3, C2C12 D6, KP-6634, and KPH2-7215 cells grown in media with or without Q, supplemented with 5 mM Pyr, 5 mM DKG, or 2 mM E and assessed after 48 h. *N* = 3 with two technical replicates. Data represent mean ± s.e.m.; *p < 0.05, **p < 0.005, ***p < 0.0005. **c** Schematic depicting the TCA cycle and production of aspartate. Filled red circles represent ^13^C atoms derived from U-^13^C-glutamine. **d** Mass isotopomer analysis of M + 4 enrichment in citrate, succinate, fumarate, and malate, represented as atom percent excess (APE), in C2C12s, KP-6634s, and KPH2-7215s cultured for 5 h with U-^13^C-glutamine. *N* = 3 with two technical replicates. Data represent mean ± s.e.m. **e** Mass isotopomer analysis of M + 4 enrichment in aspartate, represented as APE, in C2C12s, KP-6634s, and KPH2-7215s cultured for 5 h with U-^13^C-glutamine. *N* = 3 with two technical replicates. Data represent mean ± s.e.m.; *p < 0.05. **f** Total aspartate levels in C2C12s, KP-6634s, and KPH2-7215s via HPLC. *N* = 4 with two technical replicates. Data represent mean ± s.e.m.; *p < 0.05, **p < 0.005, ***p < 0.0005. *p*-Values were calculated from a two-tailed Student’s *t*-test for **a**, **b**, **d**–**f**. Source data are provided as a Source Data file.

Glutamine catabolism plays a critical role in sustaining TCA cycle activity by initial conversion to glutamate for α-ketoglutarate (α-KG) generation, an important intracellular carbon source^17,39^. To examine whether glutamine contributes to TCA anaplerosis (see Fig. 2c), glutamine-deprived KP-6634s and KPH2-7215s were supplemented with pyruvate (a glucose-derived TCA cycle donor), dimethyl 2-oxoglutarate (DKG; a cell permeable form of α-KG), or glutamate. Pyruvate, DKG, or glutamate addition effectively enhanced viability of all cells starved for glutamine (Fig. 2a, b). However, while pyruvate provided the greatest rescue of C2C12 proliferation, DKG supplementation was most effective in KP-6634s and KPH2-7215s (Fig. 2a). Notably, human HT1080s and SK-LMS-1s showed similar patterns of improved proliferation upon supplementation with pyruvate, DKG, or glutamate under glutamine starvation, although SW872 cells were unaffected by its absence (Supplementary Fig. 2A, B).

In addition to fuelling the TCA cycle, glutamate supports glutathione production to maintain redox homoeostasis^17^. To assess whether limited glutamine availability is rescued by antioxidant treatment, STS cells were cultured without glutamine and treated with *N*-acetyl cysteine (NAC). Whereas NAC supplementation failed to rescue proliferation of KP-6634 and KPH2-7215 glutamine-deprived cells, NAC treatment enhanced growth of C2C12s (Supplementary Fig. 2C). Much like KP-6634s and KPH2-7215s, NAC addition did not rescue HT1080 cell division following glutamine deprivation. However, NAC improved SK-LMS-1 proliferation upon glutamine withdrawal (Supplementary Fig. 2D), suggesting a differential glutamine usage across cells despite similar phenotypes upon glutamine withdrawal, some of which contributes to redox homoeostasis.

Collectively, our results indicate that glutamine is utilized in UPS cells as a major carbon source for the TCA cycle. To determine its intracellular fate, a stable-isotope labelled glutamine tracer (U-^13^C-glutamine) was employed to assess incorporation of glutamine-derived carbons into downstream products (Fig. 2c). Although KP-6634s and KPH2-7215s contributed similar levels of glutamine-derived carbon into the TCA cycle (citrate, succinate, fumarate, and malate) to C2C12s (Fig. 2d), incorporation into aspartate was significantly higher in KP-6634s and KPH2-7215s (Fig. 2e). Furthermore, total aspartate levels were similarly elevated in both UPS lines (Fig. 2f), much like *KP* and *KPH2* tumours compared to gastrocnemius muscle (Fig. 1d).

Glutamate, the first product of glutamine catabolism, is a primary nitrogen donor to support amino acid balance via transamination reactions^40^. We therefore also examined the contribution of glutamine nitrogens towards aspartate by using a tracer labelled on both nitrogens (^15^N_2_-glutamine; Supplementary Fig. 2E). Similar to carbon-labelled glutamine, nitrogen contribution to aspartate was increased in KP-6634s and KPH2-7215s compared to C2C12s (Supplementary Fig. 2F). Although glutamate is also used to generate alanine through transamination, ^15^N_2_ enrichment from glutamine into alanine production showed no significant difference between all three cell types (Supplementary Fig. 2G). Despite this, alanine enrichment is much higher in all three compared to aspartate enrichment, likely due to different precursors required for this transamination reaction and other amino acids present in media that may function as nitrogen sources for aspartate. The production of alanine relies on pyruvate (rather than oxaloacetate for aspartate, Supplementary Fig. 2E), which is readily available in these cells. In addition, increased aspartate levels are in line with elevated expression of glutamate-oxaloacetate transaminase 2 (GOT2), the transaminase responsible for generating aspartate, in the sarcoma lines (Supplementary Fig. 2H). Altogether, these results demonstrate that glutamine functions as a TCA cycle carbon donor and a nitrogen donor in the subsequent production of aspartate in sarcoma.

### Glutamine anaplerosis promotes nucleoside production in UPS tumours

**Fig. 3:**
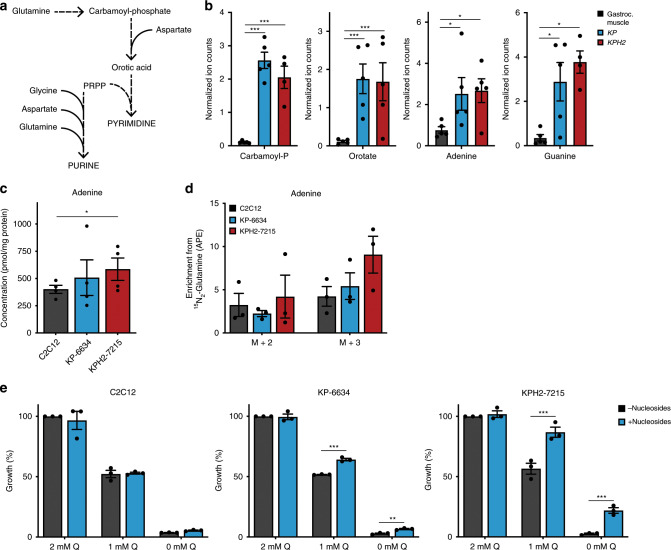
Glutamine anaplerosis promotes nucleoside production in UPS tumour cells. **a** Schematic depicting purine and pyrimidine biosynthesis. **b** Normalized ion counts for carbamoyl phosphate (carbamoyl-P), orotate, adenine, and guanine (left to right) in gastrocnemius muscle (gastroc. muscle), *KP*, and *KPH2* tumours. *N* = 4 with three technical replicates. Data represent mean ± s.d.; *p < 0.05, ***p < 0.0005. **c** Concentration of total adenine levels in C2C12s, KP-6634s, and KPH2-7215s. *N* = 3 with two technical replicates. Data represent mean ± s.e.m.; *p < 0.05. **d** Mass isotopomer analysis of M + 2 and M + 3 enrichment in adenine, represented as atoms percent excess (APE), in C2C12s, KP-6634s, and KPH2-7215s cultured for 5 h with ^15^N_2_-glutamine. *N* = 3 with two technical replicates. Data represent mean ± s.e.m. **e** Growth of C2C12s, KP-6634s, and KPH2-7215s (left to right) grown in media with 0–2 mM glutamine (Q) and supplemented with 50 μM adenosine, guanosine, cytidine, and thymidine (Nucleosides) for 72 h. *N* = 3 with three technical replicates. Data represent mean ± s.e.m.; **p < 0.005, ***p < 0.0005. *p*-Values were calculated from a two-tailed Student’s *t*-test for **b**–**e**. Source data are provided as a Source Data file.

Previous studies showed that increased glutamine anaplerosis promotes aspartate production and subsequent de novo nucleotide production to sustain cancer cell proliferation^19,20,22,41,42^. Assessment of *KP* and *KPH2* tumours for intermediates involved in nucleoside biosynthesis revealed increased levels of carbamoyl phosphate and orotate, metabolites involved in pyrimidine biosynthesis (Fig. 3a, b). In addition, increased nucleobase levels were also observed, particularly adenine and guanine (Fig. 3b). These metabolic differences between cohorts were also observed in *KPA* tumours (Supplementary Fig. 3A). To determine whether nucleotide production was the principal driver for increased glutaminolysis, sarcoma cells were cultured with ^15^N_2_-glutamine to evaluate glutamine-derived nitrogen incorporation into nucleobases, using adenine as a marker for this biosynthetic pathway (Supplementary Fig. 3B). Total adenine concentrations were elevated in KP-6634s and KPH2-7215s (Fig. 3c), and glutamine-derived nitrogen contribution to adenine was somewhat higher in these cells (Fig. 3d), although changes did not reach statistical significance.

Alongside these labelling studies, C2C12s, KP-6634s, and KPH2-7215s were exposed to varying glutamine concentrations and supplemented with four deoxyribonucleosides (adenosine, guanosine, cytidine, and thymidine). Culturing KP-6634s and KPH2-7215s under low glutamine with nucleosides increased cell proliferation, with more dramatic effects on KPH2-7215s than KP-6634s (Fig. 3e). However, despite a significant increase in cell number upon nucleoside addition to glutamine-starved cells, this was low compared to control levels of cell proliferation. Similar effects were observed in human STS cells, with HT1080s and SK-LMS-1s (sensitive to glutamine deprivation) showing enhanced cell proliferation upon nucleoside supplementation under low glutamine conditions (Supplementary Fig. 3C). Overall, we concluded that increased aspartate levels observed both in vitro and in vivo promote de novo nucleoside biosynthesis and availability to support cell proliferation, but are not the sole use of glutamine in sarcoma.

### Glutaminase 1 is highly expressed in UPS

**Fig. 4:**
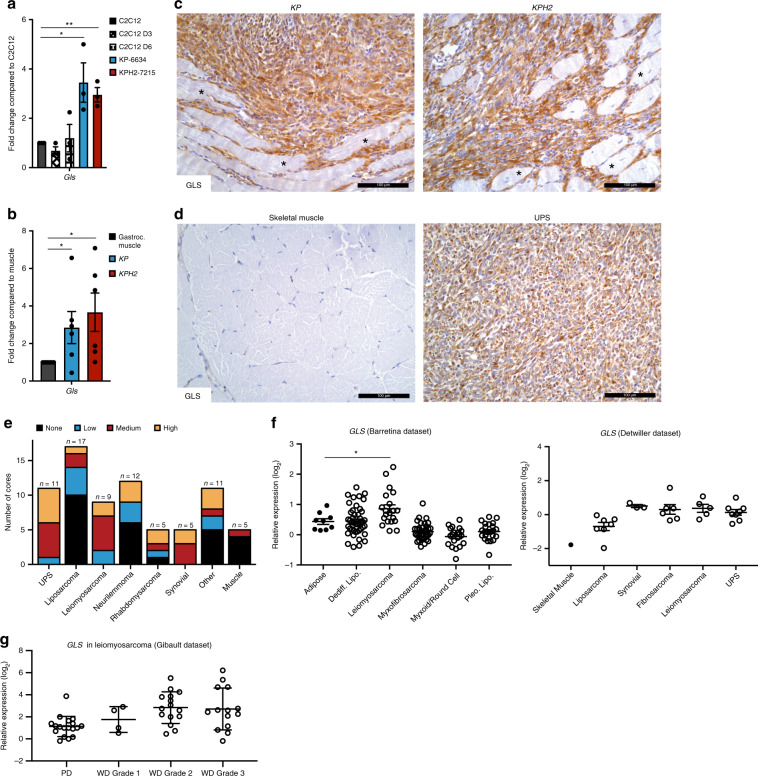
Glutaminase 1 is highly expressed in UPS. **a** *Gls* mRNA levels in C2C12, C2C12 D3, C2C12 D6, KP-6634, and KPH2-7215 cells. *N* = 3 with three technical replicates. Data represent mean ± s.e.m.; *p < 0.05, **p < 0.005. **b** *Gls* mRNA levels in gastrocnemius muscle, *KP*, and *KPH2* tumours. *N* = 5 with three technical replicates. Data represent mean ± s.e.m. *p < 0.05. **c** Representative GLS staining in *KP* (left; *n* = 8) and *KPH2* (right; *n* = 8) tumours. Asterisks indicate unstained skeletal muscle. Scale bars = 100 μm. **d** GLS staining of human skeletal muscle (left; *N* = 5) and undifferentiated pleomorphic sarcoma (UPS; right; *N* = 11). Scale bars = 100 μm. **e** Quantification of GLS staining intensity in an STS microarray. **f** *GLS* mRNA expression from Oncomine analysis of Barretina et al.^43^ (left) and Detwiller et al.^44^ (right) sarcoma patient samples datasets. Values are normalized to median-centred intensity and shown on a log_2_ scale. Dediff. lipo., dedifferentiated liposarcoma; pleo. lipo., pleomorphic liposarcoma; UPS, undifferentiated pleomorphic sarcoma; *p < 0.05. **g** *GLS* mRNA expression from Oncomine analysis of Gibault et al.^45^ leiomyosarcoma patient samples datasets. Values are normalized to median-centred intensity and shown on a log_2_ scale. PD, poorly differentiated; WD, well differentiated. *p*-Values were calculated from a two-tailed Student’s *t*-test for **a**, **b**, **f**, **g**. Source data are provided as a Source Data file.

Taken together, our in vitro data suggest that certain STS subtypes, such as UPS, fibrosarcoma, and leiomyosarcoma, are highly reliant on glutamine as a source of energy and biosynthetic anabolism. GLS catalyzes the conversion of glutamine to glutamate, is the rate-limiting enzyme for glutaminolysis, and exists in two isoforms, glutaminase 1 (GLS) and 2 (GLS2). *Gls* was the predominant isoform detected in murine tumours and cell lines (Supplementary Fig. 4A, B). In addition, KP-6634s and KPH2-7215s as well as *KP* and *KPH2* tumours showed higher *Gls* mRNA and protein compared to C2C12s and gastrocnemius muscle, respectively (Fig. 4a–c; Supplementary Fig. 4C). However, despite elevated mRNA and protein levels, the increase in GLS protein abundance is far more dramatic for unknown reasons. Human HT1080s, SK-LMS-1s, SK-UT-1Bs, CCL-136s, LPS246s, and T778s exhibited high GLS expression (GAC isoform; * band) compared to SW872s (Supplementary Fig. 4D). Notably, LPS246s and T778s also showed high levels of the KGA isoform (*** band) compared to the other cell lines. Altogether, this suggests different mechanisms for resistance to glutamine deprivation observed in LPS246s and SW872s (Supplementary Fig. 1G, H). Therefore, liposarcomas may rely on other metabolic pathways for their energetic and anabolic purposes that differentiate them from fibrosarcoma, leiomyosarcoma, and rhabdomyosarcoma cells.

These observations were further reflected by increased GLS abundance in UPS tissues compared to skeletal muscle (Fig. 4d). Furthermore, of all biopsies available, a majority stained medium (38.0%) or high (32.9%) for GLS using immunohistochemistry (Supplementary Fig. 4E, F). To determine whether GLS was elevated in other STS subtypes, a variety of STS tumours was examined. Similarly, UPS stained medium (45.5%) and high (45.5%) for GLS, while leiomyosarcoma (medium, 55.6%; high, 22.2%) and synovial sarcoma (medium, 60%; high, 40%) were also positive for GLS. Other STSs (e.g. liposarcomas and neurilemmomas) exhibited little to no GLS expression, indicating that in vitro differences correlated with GLS-expressing STS subtypes (Fig. 4e; Supplementary Fig. 4G). This was further confirmed when publicly available microarrays of human STS were queried. Datasets originally published by Barretina et al.^43^ and Detwiller et al.^44^ demonstrated that *GLS* expression is higher in leiomyosarcomas compared to adipose tissue, and UPS samples compared to normal skeletal muscle (Fig. 4f). Interestingly, variable *GLS* mRNA expression across STS subtypes (Fig. 4f) was also noted for protein levels in our tissue microarray and human cell line analyses (Fig. 4e; Supplementary Fig. 4D–G). For example, liposarcoma patient samples exhibited similar GLS expression levels. Furthermore, GLS2 expression was low or similar to normal tissue (Supplementary Fig. 4H). While survival data were not available in the aforementioned datasets, Gibault et al.^45^ provided *GLS* expression for leiomyosarcoma patients based on disease grade. Poorly differentiated (PD) leiomyosarcoma showed lowest *GLS* levels, which increased with grade in well-differentiated (WD) patients (Fig. 4g). In summary, GLS is elevated in multiple STS subtypes and may increase with disease progression, and higher GLS levels correlate with subtypes most sensitive to glutamine metabolism modulation.

### GLS inhibition with CB-839 targets UPS cells

**Fig. 5:**
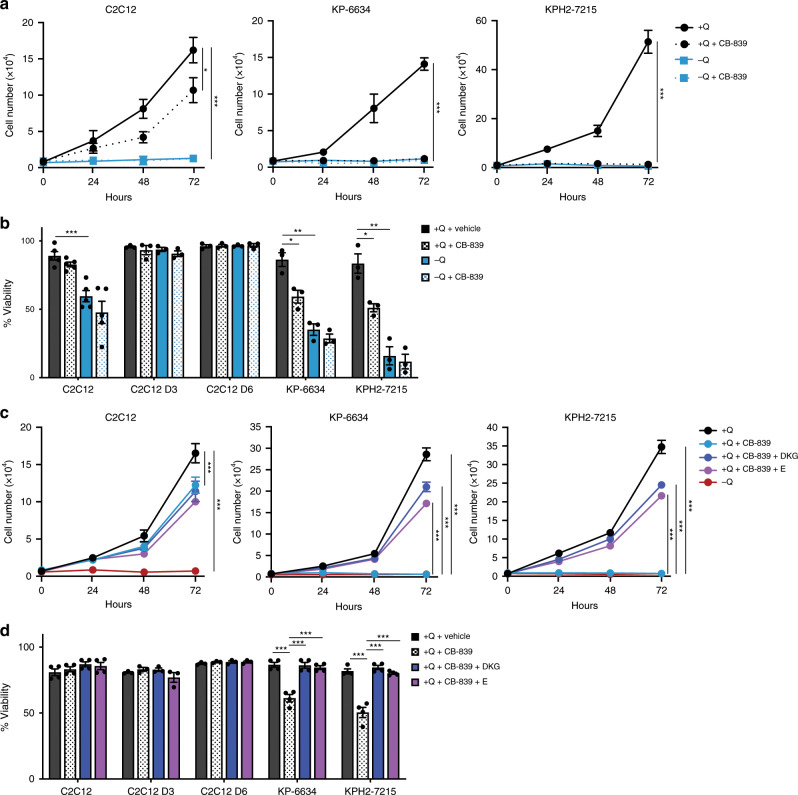
Glutaminase inhibition with CB-839 and effects on UPS cells. **a** Proliferation of C2C12s, KP-6634s, and KPH2-7215s (left to right) grown in media with or without glutamine (Q) and treated with 1 μM CB-839. *N* = 3 with three technical replicates. Data represent mean ± s.e.m.; *p < 0.05, ***p < 0.0005. **b** Viability of C2C12, C2C12 D3, C2C12 D6, KP-6634, and KPH2-7215 cells grown in media with or without Q, treated with 1 μM CB-839 and assessed after 48 h. *N* = 3 with two technical replicates. Data represent mean ± s.e.m.; *p < 0.05, **p < 0.005, ***p < 0.0005. **c** Proliferation of C2C12s, KP-6634s, and KPH2-7215s (left to right) grown in media with or without Q, treated with 1 μM CB-839, and 5 mM dimethyl 2-oxoglutarate (DKG) or 2 mM glutamate (E). *N* = 3 with three technical replicates. Data represent mean ± s.e.m.; ***p < 0.0005. **d** Viability of C2C12, C2C12 D3, C2C12 D6, KP-6634, and KPH2-7215 cells grown in media with or without Q, treated with 1 μM CB-839, 5 mM DKG, or 2 mM E, and assessed after 48 h. *N* = 3 with two technical replicates. Data represent mean ± s.e.m.; ***p < 0.0005. *p*-Values were calculated from a two-tailed Student’s *t*-test for **a**–**d**. Source data are provided as a Source Data file.

Having established that UPS and glutamine-sensitive STS subtypes express higher GLS levels, we determined whether a small molecule GLS inhibitor, Telaglenastat (CB-839), currently in clinical trials for multiple cancer types, is effective in causing UPS and STS cell death. Consistent with glutamine deficiency, KP-6634s, KPH2-7215s, HT1080s, SK-LMS-1s, SK-UT-1Bs, CCL-136s, and T778s were highly responsive to CB-839 treatment, i.e. GLS inhibition mimicked glutamine starvation (Fig. 5a, b; Supplementary Fig. 5A, B). Much like glutamine starvation, only T778 cell proliferation and not viability was affected (Fig. 5a, b; Supplementary Fig. 5A, B). Despite their sensitivity to glutamine withdrawal, CB-839 only mildly decreased C2C12 cell proliferation with no effect on cell viability (Fig. 5a), possibly due to differential glutamine usage. CB-839 treatment had no effect on differentiated C2C12s (Fig. 5b) and human LPS246s and SW872s (Supplementary Fig. 5A, B), indicating a lack of glutamine dependency. Furthermore, CB-839 combined with glutamine starvation had no additive effects on cell proliferation and viability, demonstrating that CB-839 is highly specific for GLS activity. This sensitivity to CB-839 is maintained under long-term proliferation assays, where following 8 days of culture, sarcoma lines (KP-6634s, KH2-7215s, and HT1080s) remain unable to grow in CB-839-treated media, while C2C12s have slower proliferation rates but are still viable (Supplementary Fig. 5C). This may be attributed to overt cell death in the sarcoma lines preventing the outgrowth of resistant cell populations. Altogether, these results demonstrate that glutamine deprivation inhibits in vitro growth and viability of multiple STS cell types, but much like glutamine sensitivity, not all subtypes behave similarly.

By inhibiting GLS, downstream production of glutamate and, subsequently, α-KG should be decreased. Therefore, we treated mouse and human STS cells with CB-839 combined with DKG or glutamate supplementation to determine whether these additional metabolites reversed CB-839 effects on cell growth and survival. Importantly, glutamate or DKG addition rescued cell proliferation and viability upon GLS inhibition in KP-6634s, KPH2-7215s, HT1080s, and SK-LMS-1s (Fig. 5c, d, Supplementary Fig. 5D, E). In addition, CB-839 effects were not reversed by the antioxidant NAC in KP-6634s and KPH2-7215s, although decreased C2C12 cell proliferation was slightly improved by concurrent NAC treatment (Supplementary Fig. 5F). While NAC supplementation did not change CB-839 effects on HT1080s, NAC improved CB-839-treated SK-LMS-1 cell proliferation (Supplementary Fig. 5G). Increased sensitivity to both glutamine deprivation and GLS inhibition suggests that UPS and highly GLS^+^ STS subtypes depend on glutamine-derived glutamate for energetic and biosynthetic demands rather than redox homoeostasis and antioxidant production, which may be achieved through additional mechanisms.

### GLS inhibition effectively blunts in vivo UPS growth

**Fig. 6:**
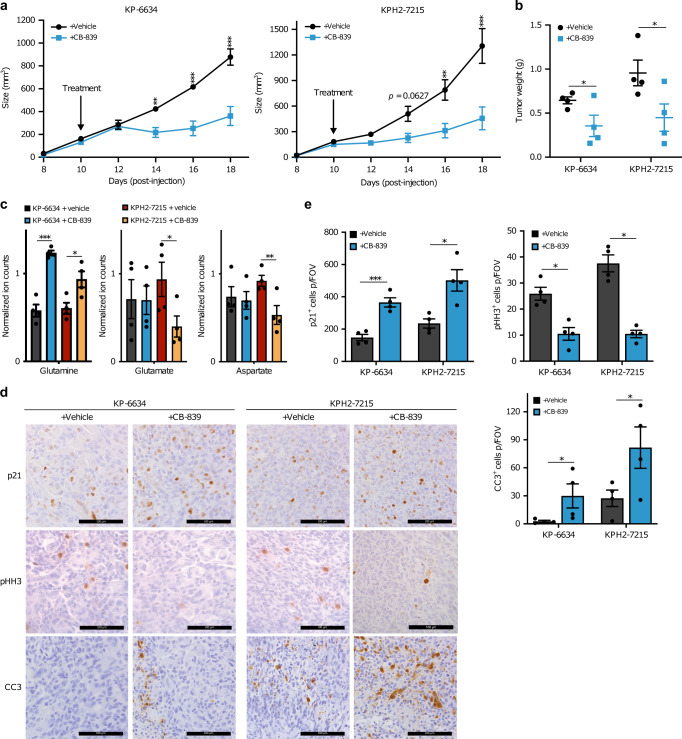
Glutaminase inhibition effectively blunts UPS allograft growth. **a** Tumour size of subcutaneous KP-6634 (left), KPH2-7215 (right) allografts in vehicle (*n* = 4) or CB-839-treated mice (*n* = 4). Mice were treated once tumours reached ∼100 mm^3^ at ~10 days post-injection. Data represent mean ± s.d.; **p < 0.005, ***p < 0.0005. **b** Tumour weights from vehicle- and CB-839-treated KP-6634 and KPH2-7215 allograft mice (*n* = 4). Error bars are s.d.; *p < 0.05. **c** Normalized ion counts for glutamine, glutamate, and aspartate (left to right) in KP-6634 and KPH2-7215 allograft tumours from vehicle- and CB-839-treated animals. *N* = 4 with two technical replicates. Data represent mean ± s.d.; *p < 0.05, **p < 0.005, ***p < 0.0005. **d** Representative immunohistochemistry staining for p21 (top row), phospho-histone H3 (middle row; pHH3), and cleaved-caspase 3 (bottom row; CC3) in KP-6634 and KPH2-7215 allografts of vehicle- (*n* = 4) and CB-839-treated (*n* = 4) animals. Scale bars = 100 μm. **e** Quantification of p21^+^ (top left), pHH3^+^ (top right), and CC3^+^ (bottom right) cells per field of vision (FOV) in vehicle- and CB-839-treated animals. *N* = 4 with five images taken per section. Data represent mean ± s.e.m.; *p < 0.05, ***p < 0.0005. *p*-Values were calculated from a two-tailed Student’s *t*-test for **a**–**c**, **e**. Source data are provided as a Source Data file.

Our findings suggest that GLS inhibition suppresses proliferation and viability of tumour-derived UPS, HT1080, and SK-LMS-1 cells in vitro. We then tested CB-839’s ability to inhibit UPS tumour growth in vivo using allografted KP-6634 and KPH2-7215 cells. When tumours reached ~100 mm^3^, animals were treated with vehicle or CB-839 twice daily as described^30^. CB-839 administration did not affect mouse weights (Supplementary Fig. 6A), but significantly reduced tumour growth (Fig. 6a) and final tumour mass in both KP-6634 and KPH2-7215 allografts (Fig. 6b). As noted previously, KPH2-7215 allografts were larger than KP-6634 allografts, but also more sensitive to CB-839 treatment. Tumours from CB-839-treated mice showed increased glutamine levels with concurrent decreased glutamate and aspartate levels, confirming that CB-839 effectively blocks glutamine breakdown and production of downstream metabolites (Fig. 6c). Further histological analyses revealed increased cell cycle arrest (p21^+^ cells), decreased cell proliferation (phospho-histone H3^+^ cells), and increased cell death (cleaved-caspase 3^+^ cells) in tumours from CB-839-treated animals (Fig. 6d, e).

To assess whether human cell lines also remained sensitive to CB-839 in vivo, we treated HT1080 xenograft-bearing animals with CB-839. Consistent with allograft studies, CB-839 administration did not alter animal weights (Supplementary Fig. 6B), and significantly reduced tumour growth (Supplementary Fig. 6C) and final tumour weights (Supplementary Fig. 6D). Histological staining showed decreased numbers of proliferative cells and increased cell death and cell cycle arrest (Supplementary Fig. 6E, F), much like CB-839-treated allografts.

**Fig. 7:**
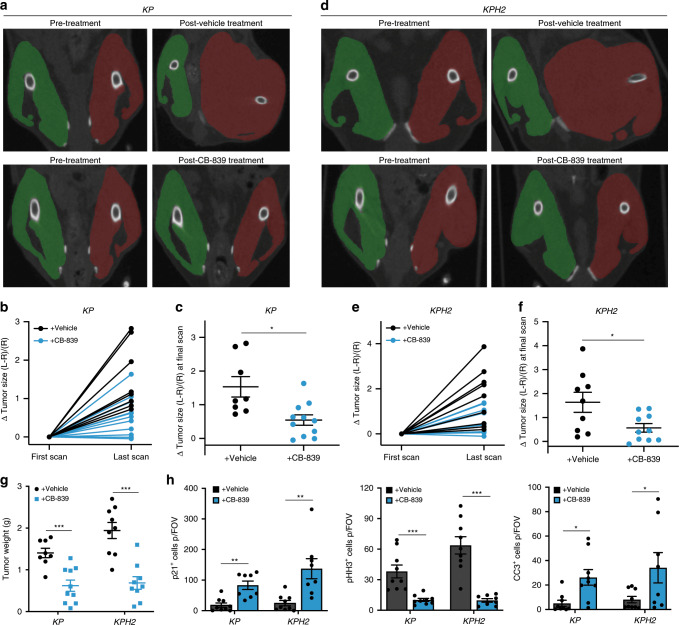
CB-839 is effective in an autochthonous UPS model. **a** Representative computed tomography (CT) scans of *KP* mice pre- and post-treatment with vehicle (*n* = 8) or CB-839 (*n* = 10) following up to 16 days of treatment. Right control (green) and left tumour-bearing (red) limbs are highlighted. **b** Change in tumour size as quantified from the relative difference in size of the two limbs normalized to the control limb in *KP* vehicle- (*n* = 8) or CB-839-treated (*n* = 10) mice. **c** Change in tumour size at final CT scan following vehicle (*n* = 8) or CB-839 treatment (*n* = 10) in *KP* mice. Error bars are ± s.d.; *p < 0.05. **d** Representative CT scans of *KPH2* mice pre- and post-treatment with vehicle (*n* = 9) or CB-839 (*n* = 10) following up to 21 days of treatment. Right control (green) and left tumour-bearing (red) limbs are highlighted. **e** Change in tumour size as quantified from the relative difference in size of the two limbs normalized to the control limb in *KPH2* vehicle- (*n* = 9) or CB-839-treated (*n* = 10) mice. **f** Change in tumour size at final CT scan following vehicle (*n* = 9) or CB-839 treatment (*n* = 10) in *KPH2* mice. Error bars are ± s.d.; *p < 0.05. **g** Tumour weights from vehicle- and CB-839-treated *KP* (vehicle *n* = 8; CB-839 *n* = 10) and *KPH2* (vehicle *n* = 9; CB-839 *n* = 10) mice. Error bars are ± s.e.m.; ***p < 0.0005. **h** Quantification of p21^+^, phospho-histone H3^+^ (pHH3), and cleaved-caspase 3^+^ (CC3) cells per field of vision (FOV) in vehicle- and CB-839-treated animals (left to right). *N* = 8 with five images taken per section. Data represent mean ± s.e.m.; *p < 0.05, **p < 0.005, ***p < 0.0005. *p*-Values were calculated from a two-tailed Student’s *t*-test for **c**, **f**–**h**. Source data are provided as a Source Data file.

Most importantly, we evaluated CB-839 efficacy in an autochthonous STS model using *KP* and *KPH2* mice. After initiating tumours with AdCre injection, we performed bi-weekly computed tomography (CT) scans of mouse lower limbs to track tumour growth. As minor limb size differences emerged, animals were treated with vehicle or CB-839 for ~2–3 weeks. Once again, CB-839 treatment did not impact mouse weights (Supplementary Fig. 7A, B). However, in marked contrast to previous in vivo studies^33,34^, CB-839 administration to *KP* and *KPH2* animals substantially inhibited tumour growth, as calculated from the difference in the muscular compartment of tumour-bearing limbs (red) relative to control limbs (green) (Fig. 7a–f). CT-quantified tumour size strongly correlated with tumour mass, and CB-839 treatment significantly reduced final tumour weight in *KP* and *KPH2* animals compared to vehicle-treated mice (Fig. 7g), with stronger effects observed in *KPH2* mice, much like allograft studies (Fig. 6a, b). Histological analyses showed CB-839 increased cell cycle arrest, decreased cell proliferation, and increased cell death in established tumours (Fig. 7h; Supplementary Fig. 7C).

In summary, *GLS* upregulation promotes glutamine utilization to sustain the TCA cycle and maintain energetic as well as biosynthetic demands in sarcoma cell growth and proliferation. Surprisingly, CB-839 effectively blocked UPS growth in a transgenic mouse model, thereby successfully recapitulating our in vitro data. Taken together, our studies expand the potential clinical use of GLS inhibition to include UPS and other high GLS-expressing STS subtypes.

## Discussion

Current therapeutic approaches for unresectable STS have a low response rate, with genetically diverse subtypes representing a major obstacle to developing better treatment regimens^1,5^. Furthermore, relatively low incidence has left sarcoma an understudied disease. By identifying therapeutic biomarkers for STS subtypes, newer targeted and combinatorial therapies could successfully stratify patient populations into those most likely to benefit. Tumorigenesis requires cancer cells to increase their metabolic output to support tumour growth. Glutamine fuels cellular bioenergetics and supports multiple biosynthetic processes, making it an important nutrient for highly proliferative cells. Specifically, glutamine’s carbon backbone can be utilized for the production of TCA cycle intermediates, amino acids, and other metabolites^18–20,22,27^, while glutamine-derived nitrogen also promotes nucleotide biosynthesis^18^.

We show murine UPS and human fibrosarcoma and leiomyosarcoma cell proliferation in vitro is dependent on glutamine-derived glutamate, supporting central carbon metabolism, aspartate production, nucleotide biosynthesis, and subsequently, cell division (Figs. 1–3). This metabolic dependency is primarily due to defective TCA cycle anaplerosis upon glutamine deprivation (Figs. 2, 5). Although human STSs show significant heterogeneity of GLS expression (Supplementary Fig. 4D, E), we noted a striking enrichment in UPS (Fig. 4e). GLS is transcriptionally^17^, post-transcriptionally^46^, and post-translationally^47^ regulated. We previously established that HIF-2α loss in UPS tumours leads to enhanced mechanistic target of rapamycin complex 1 (mTORC1) signalling^38^, a key pathway in nutrient sensing, cellular growth, and ribosomal biogenesis. mTORC1 is often deregulated in multiple cancers and its activation promotes anaplerotic glutamine entry into the TCA cycle^48^. Moreover, mTORC1 positively impacts *GLS* expression through S6K1-dependent regulation of c-Myc^49^. Sequencing UPS patient samples identified mutated genes correlated with cell cycle, PI3K/mTOR, and RAS/MAPK signalling pathways^50^, suggesting that *GLS* may be mTORC1/Myc-driven in UPS. However, further studies are necessary to define critical GLS regulatory pathways in sarcomas.

Given that GLS expression supports sarcomagenesis, we evaluated GLS inhibitors and determined that Telaglenastat (CB-839; a small molecule inhibitor in phase I and II clinical trials^32^) effectively blocks GLS activity and sarcoma growth (Figs. 5–7). CB-839 has now been assessed pre-clinically in vitro and in vivo for the treatment of a variety of cancer types^17,30,33,51^. While CB-839 was previously tested in one STS subset, *NF1*-associated soft tissue malignancies, this was limited to in vitro assays and a xenograft study^52^. CB-839 has also been previously examined in *KP* models of lung cancer and PDAC^33,34^. Despite similar responses to those we observed for STS in vitro, CB-839 was not effective in vivo with pyruvate metabolism being more important in lung cancer and adaptive metabolic rewiring conferring resistance to CB-839 in PDAC. Our study shows CB-839 treatment effectively reduces autochthonous UPS tumours in vivo. Most interesting is the differential response to CB-839 across all three *KP* mouse models following the same treatment regimen. Skeletal muscle accounts for ~70% of endogenous glutamine production in humans^53^. With such a quantitively large contribution, we hypothesize that the effectiveness of GLS inhibition in UPS is due to high glutamine availability produced by surrounding muscle tissue. This easy access to glutamine from neighbouring organs likely makes limb sarcomas (e.g. UPS and leiomyosarcoma) reliant on exogenous sources, sensitizing them to CB-839, in contrast to lung and PDAC, or STS subtypes that do not express GLS, such as liposarcoma.

In addition to varied responses in *KP* models, glutamine dependency and CB-839 sensitivity do not appear to be solely reliant on mutant Ras expression, with substantial genetic heterogeneity across all the cell lines tested (HT-1080, *NRAS* mutation; CCL-16, *NRAS* mutation; SK-LMS-1s, no Ras mutation; SK-UT-1B, no Ras mutation; LPS246, *MDM2* amplification; T778, *MDM2*, *CDK4*, and *HMGA2* amplification; SW872, *BRAF* mutation). Furthermore, based on The Cancer Genome Atlas (TCGA) analysis^5^, dedifferentiated liposarcomas exhibit highly recurrent copy-number gains of *MDM2*, *CDK4*, and *HMGA2*. For leiomyosarcomas, deletions or mutations of *TP53*, *RB1*, and *PTEN* were found. Analysis in UPS samples demonstrated high-level amplification of *CCNE1*, *VGLL3*, and *YAP1*. Other studies have shown that GLS sensitivity does not completely correlate with GLS expression^54^, similar to our findings. While the majority of glutamine-dependent and CB-839-sensitive cell lines express GLS, LPS246s are resistant to glutamine starvation and CB-839 treatment despite having comparable GLS levels (unlike SW872s; Supplementary Fig. 4D). Therefore, liposarcomas may rely on other metabolic pathways for energetic and anabolic purposes, possibly by using glucose or lipids to support the TCA cycle, thereby allowing cells to overcome GLS disruption. Altogether, the response to GLS inhibition remains highly dependent on cell of origin and surrounding tissue to dictate tumour metabolism and sometimes the adaptive mechanisms that mitigate effects of metabolic drugs, like CB-839.

Single-agent inhibition is unlikely to provide durable benefit in controlling advanced disease^17^ and recent studies report CB-839 synergizes with β-lapachone in PDAC^55^ and BCL-2 inhibition in acute myeloid leukaemia^31^. Due to numerous genomic alterations in STSs^2,5,12^, DNA repair pathways offer an interesting target alongside GLS inhibition. For example, GLS inhibition increases radiotherapy response in lung tumour xenografts^56^ and synergy with poly(ADP-ribose) polymerase (PARP) inhibition was investigated in renal carcinoma cells, where GLS inhibitors led to nucleoside depletion and DNA replication stress. Combination of GLS1 inhibitors with PARP inhibitors (PARP_i_) suppressed cell growth^57^. Collectively, these findings emphasize GLS’ role in nucleotide synthesis, where inhibition leads to DNA damage and replication arrest. DNA damage response inhibitors, including PARP_i_, have shown promising results, particularly in Ewing sarcoma, an STS affecting adolescents and young adults bearing *EWS-FLI1* or *EWS-ERG* genomic fusions with the EWS-FLI1 oncoprotein increasing PARP expression^2,58^. Pre-clinical evidence for PARP_i_ in combination with chemotherapy or radiotherapy appears promising^59–61^, suggesting PARP_i_ are best combined with DNA damage-inducing treatments, such as GLS inhibition.

Although not all nucleobases showed consistent changes, adenine was increased in sarcoma cell lines and tumours compared to C2C12s and muscle tissue, respectively (Fig. 3b-d). In addition to supporting cell proliferation, adenine maintains synthesis of nicotinamide adenine dinucleotide (NAD), a redox cofactor and substrate for signalling enzymes, including PARP^62^. NAD is generated from nicotinamide and consumed largely by PARPs and sirtuins in vitro, and selectively generated in vivo from tryptophan in the liver^63^. Tryptophan was elevated in the sarcoma tumours compared to muscle (Supplementary Fig. 1C-G) and nicotinamide metabolism was highlighted in metabolic pathway enrichment analyses (Fig. 1c). Based on this, we speculate that CB-839 combined with PARP_i_ could have synergistic effects in GLS-expressing STSs; however, further studies are required to test this directly. Notably, a recent study using the glutamine antagonist prodrug JHU083 in tumour-bearing mice suppressed oxidative and glycolytic metabolism of cancer cells. In contrast, effector T cells responded to glutamine antagonism by upregulating oxidative metabolism and adopting a long-lived, highly activated phenotype, disrupting the nutrient milieu of the tumour microenvironment^64^. Our findings and those using JHU083 underscore the therapeutic potential of targeting glutamine metabolism.

Overall, the notion of combining standard chemotherapies, such as PARP_i_, or other DNA damaging agents, such as radiation and doxorubicin^10^, first line therapies in STS, with CB-839 is a potentially suitable treatment strategy. Furthermore, we suggest GLS inhibition will be most effective against sarcomas expressing high GLS levels, making it a potential biomarker to further stratify sarcoma patients.

## Methods

### Animals

Tumours were generated in 8 weeks or older male and female *LSL-Kras*^*G12D/+*^*;Trp53*^*fl/fl*^ (*KP*^35^), *LSL-Kras*^*G12D/+*^*;Trp53*^*fl/fl*^*;Epas1*^*fl/fl*^ (*KPH2*^38^), and *LSL-Kras*^*G12D/+*^*;Trp53*^*fl/fl*^*;Arnt*^*fl/fl*^ (*KPA*^38^*)* mice by injecting adenovirus expressing Cre-recombinase (AdCre) into the hindlimb musculature. Animals were sacrificed when tumours reached maximal permissible size. For subcutaneous xenografts and allografts, 1 × 10^6^ cells were injected in 200 μL of 1:1 PBS:Matrigel (Corning) into the flanks of female Balb/c nu/nu mice (Charles River Laboratories). Transplantable tumours were measured every other day by electronic caliper measurements. Animals were euthanized by CO_2_ inhalation at endpoint. Transplantable tumour volumes were calculated using the formula volume = (length × (width)^2^)/2. Sample size for each experiment was estimated using the formula *n* = ((*zα*/2*σ*)/*E*)^2^, with *α* = 0.05, and *σ* and *E* based off initial *KP* experiments with vehicle or CB-839 treatment. No inclusion or exclusion criteria parameters were used and all experimental animals were included in analyses. Animal wellbeing was monitored by certified veterinary staff. All mouse experiments were performed according to National Institutes of Health guidelines and received ethical approval by the University of Pennsylvania Institutional Animal Care and Use Committee.

### In vivo drug treatment

Once transplantable tumours were established (~100 mm^3^) or hindlimb differences were observed by CT imaging (~4–6 weeks post-injection), animals were randomized into two groups and treated with vehicle (25% (w/v) hydroxypropyl-β-cyclodextrin in 10 mmol/L citrate (pH 2.0)) or 200 mg/kg Telaglenastat (CB-839; Calithera Biosciences) twice daily administered through oral gavage^30^. Telaglenastat was formulated at 20 mg/mL for a final dosing volume of 10 mL/kg. Researchers were not blinded to the experimental groups during in vivo treatments.

### CT imaging and analysis

Computer tomography (CT) images were generated using an MiLabs U-CT scanner. Scan settings were 50 kVp, 240 μA with 75 ms exposure time per projection and 480 projections were taken per scan. Tumour volumes were quantified using ITK-SNAP 3.6.0. Given the similar attenuation of limb muscle and tumour, tumour size was assessed as the difference in the muscular compartment of the treated (L) and control limb (R), normalized to the control limb (L–R)/R. The muscular compartment was quantified by manual segmentation of a coronal slice that traversed the mid-femur of both limbs. Change in tumour size was assessed relative to the tumour size measured at the first imaging timepoint. Researchers were blinded to the experimental groups during CT imaging analysis.

### Cell culture

HT1080, SK-LMS-1, SK-UT-1B, CCL-136, SW872, 94T778 (T778), and C2C12 cells were purchased from ATCC and authenticated by the manufacturer by short tandem repeat. LPS246 cells were kindly provided by Dr. Dina Lev (MD Anderson Cancer Center). KP-6634s and KPH2-7215s were derived from UPS mouse tumours^38^. MMSCs were purchased from Cyagen and authenticated by the manufacturer. All cells were cultured in DMEM (Thermo Fisher Scientific) unless otherwise noted. HT1080, SK-LMS-1, SW872, KP-6634, and KPH2-7215 cells were maintained in DMEM supplemented with 10% FBS (GEMINI) and 2 mM l-glutamine (Thermo Fisher Scientific). C2C12s were maintained in DMEM supplemented with 20% FBS with 2 mM l-glutamine. MMSCs were maintained in Mesenchymal Stem Cell Growth Medium (Cyagen). C2C12s were differentiated by changing media to DMEM with 5% horse serum (Thermo Fisher Scientific) and 2 mM l-glutamine. Cells were maintained in 37 °C, 5% CO_2_ humidified incubators. All cells are routinely confirmed as Mycoplasma negative (MycoAlert; tested every 3 months).

### Proliferation assays

Cells (1 × 10^4^ p/mL for 72 h assay, 1 × 10^3^ p/mL for 8-day assay) were seeded in maintenance media and allowed to adhere overnight before receiving assay media using glucose/glutamine-free DMEM (Thermo Fisher Scientific) with 10% dialyzed FBS (dFBS; GEMINI). Media were supplemented with indicated compounds (Sigma-Aldrich, unless noted): 5 mM glucose, 2 mM l-glutamine (Thermo Fisher Scientific), 5 mM pyruvate, 5 mM dimethyl-2-oxoglutarate, 2 mM glutamate, 2 mM NAC, or 1 μM Telaglenastat (CB-839; Calithera Biosciences). Nucleoside supplementation consisted of 50 μM adenosine, cytidine, guanosine, thymidine, and uridine each. Cells were counted by trypan blue exclusion on a Countess II (Thermo Fisher Scientific).

### Viability assays

Cells (1 × 10^5^ p/mL) were seeded in maintenance media in 6-well plates and allowed to adhere overnight before receiving assay media as described above. After 48 h, cell viability was assessed by FITC-Annexin V/PI Kit (BD Biosciences; Cat. #556547) according to the manufacturer’s instructions. Flow cytometry was performed using a BD FACSCalibur. Flow plots exemplifying gating strategy can be found in Supplementary Figure 8.

### Immunoblots

Cells were harvested in lysis buffer (40 mM HEPES (pH 7.4), 2 mM EDTA, 10 mM pyrophosphate, 10 mM glycerophosphate, 1% Triton X-100) with Complete Ultra protease/phosphatase inhibitor (Roche; Cat. #05892791001). Samples were centrifuged at 20,000×*g* for 15 min at 4 °C. Protein lysates were resolved by Tris-Glycine SDS-PAGE and transferred to nitrocellulose membranes (Biorad; Cat. #162-0115). Membranes were incubated with primary antibodies overnight at 4 °C diluted in TBST supplemented with 5% bovine serum albumin (BSA): GLS (1:500; Abcam; Cat. #ab156876) and HSP90 (1:1000; Cell Signaling; Cat. #4874). Primary antibodies were detected with horseradish peroxidase-conjugated secondary antibodies followed by exposure to SuperSignal West Femto Maximum Sensitivity Substrate ECL reagents (Thermo Fisher Scientific; Cat. #34095).

### Gene expression analyses

RNA extraction was done using the RNeasy kit (QIAGEN; Cat. #74134) following manufacturer’s instructions. cDNA was synthesized from 2 μg of RNA using the High Capacity RNA-to-cDNA kit (Thermo Fisher Scientific; Cat. #4387406) following manufacturer’s instructions with a C1000 Thermal Cycler (Bio-Rad). qRT-PCR was performed with 2 μL cDNA and Taqman 2X Universal PCR Master Mix (Thermo Fisher Scientific) using a ViiA7 Real-Time PCR System (Applied Biosystems). Taqman primers were obtained from Life Technologies: *Gls* (Mm01257297_m1), *Gls2* (Mm01164862_m1), *GLS* (Hs00248163_m1), and *GLS2* (Hs00998733_m1). Gene expression was quantified relative to the housekeeping genes *Rn18s* (Mm03928990_g1) or *RNA18S* (Hs03928985_G1).

### Immunohistochemistry

Slides were deparaffinized, rehydrated, and quenched in 0.6% hydrogen peroxide/methanol for 15 min. For antigen retrieval, slides were boiled for 20 min in 10 mM sodium citrate (pH 6.0). Sections were blocked with 5% serum/1% BSA/0.5% Tween-20 for 1 h and incubated with primary antibodies diluted in blocking buffer overnight at 4 °C. Following primary antibody, slides were incubated with biotinylated secondary antibodies followed by ABC solution (Vector Laboratories) and developed with 3,3′-diaminobenzidine (Vector Laboratories). Slides were counterstained with haematoxylin, dehydrated, and mounted with Permount (ThermoFisher Scientific). STS (SO801) and UPS (SO802) tissue microarray slides were purchased from US Biomax. Primary antibodies used: cleaved-caspase 3 (1:200; Cell Signaling; Cat. #9661), GLS (1:200; Abcam; Cat. #ab156876), p21 (1:500; Abcam; Cat. #ab188224), phospho-Histone H3 (1:200; Cell Signaling; Cat. #3377).

### Stable isotope metabolite tracing

For labelling studies, C2C12s, KP-6634s, and KPH2-7215s were plated in 10 cm dishes and allowed to adhere overnight. Labelling media consisted of glucose/glutamine-free DMEM (Thermo Fisher Scientific) with 10% dFBS and 5 mM (unlabelled) glucose. In addition, 2 mM U-^13^C-glutamine (Cambridge Isotopes; Cat. #CLM-1822-H-PK) or 2 mM ^15^N_2_-glutamine (Cambridge Isotopes; Cat. #NLM-1328-PK) was added. Cells were incubated in labelling media for 5 h before GC-MS measurement of ^13^C enrichment in TCA cycle intermediates and/or ^15^N enrichment in amino acids were performed as previously described^65–67^. Briefly, cells were washed twice with PBS, metabolites were extracted with 4% perchloric acid, and cell extracts were neutralized with KOH. Neutralized extracts were subjected to either AG-1 column (100-200 mesh, 0.5 × 2.5 cm; Biorad) for enriching the organic acids, or AG-50 (100-200 mesh, 0.5 × 2.5 cm; Biorad) for enriching amino acids. The collected samples were then converted to *t*-butyldimethylsilyl derivatives^65–67^.

In studies with ^13^C-labelled glutamine, isotopic enrichment in ^13^C-glutamate isotopomers was monitored using ions at *m*/*z* 432, 433, 434, 435, 436, and 437 for M0, M1, M2, M3, M4, and M5 (containing one to five ^13^C atoms above M0, the natural abundance), respectively. Isotopic enrichment in ^13^C aspartate isotopomers was monitored using ions at *m*/*z* 418, 419, 420, 421, and 422 for M0, M1, M2, M3, and M4 (containing one to four ^13^C atoms above M0, the natural abundance), respectively. Isotopic enrichment in ^13^C malate isotopomers was evaluated using ions at *m*/*z* 419, 420, 421, 422, and 423 for M0, M1, M2, M3, and M4 (containing one to four ^13^C atoms above natural abundance), respectively. Isotopic enrichment in ^13^C fumarate isotopomers was monitored using ions at *m*/*z* 287, 288, 289, 290, and 291 for M0, M1, M2, M3, and M4 (containing one to four ^13^C atoms above natural abundance), respectively. Isotopic enrichment in ^13^C succinate isotopomers was evaluated using ions at *m*/*z* 289, 290, 291, 292, and 293 for M0, M1, M2, M3, and M4 (containing one to four ^13^C atoms above natural abundance), respectively, and ^13^C enrichment in ^13^C citrate isotopomers was assayed using ions at *m*/*z* 459, 460, 461, 462, 463, 464, and 465 for M0, M1, M2, M3, M4, M5, and M6 (containing one to six ^13^C atoms above natural abundance), respectively. In experiments with ^15^N-labelled glutamine, isotopic enrichment in alanine was evaluated using ions at *m*/*z* 232 and 233 for M0 and M1, respectively. In glutamate, *m*/*z* 432 and 433 for M0 and M1, respectively, and aspartate, *m*/*z* 418 and 419 for M0 and M1, respectively.

The LC-MS/MS Agilent Infinity 1260 Triple Quad 6410B system measured total levels and ^15^N-labelled adenine production in pH-neutralized cell extracts. For ^15^N-enrichment in adenine, single ions were monitored: 136 (M), 137 (M1), 138 (M2), and 139 (M3). Total adenine was determined by spiking the sample with a known concentration of internal standard (IS), E-aminocaproic acid. Measurements were performed with LC-MS/MS, using MRM ions-pairing 136-119 for adenine and 132-114 for IS.

### Metabolomic analysis

For steady-state metabolomic analysis, 300 μL of 2:1 methanol/chloroform was added to ∼50 mg tissue (for normalization based on tissue weight) and homogenized using the TissuLyser II (Qiagen). Next, 100 μL of water and chloroform were added and samples were vortexed and centrifuged at 18,787 × *g* for 7 min at 4 °C. The upper layer containing polar metabolites was transferred and dried in a speed vacuum. Dried samples were reconstituted in 100 μL of 1:1 acetonitrile/water, vortexed, and spun down at 18,787 × *g* for 7 min at 4 °C. For UPLC-MS analysis, conditions were used as previously described^68,69^. In short, in analytical triplicate, 5 μL of the sample was injected on a XBridge BEH amide column through an Acquity H-class UPLC system (Waters Corporation). MS was done using a Waters Xevo-TQS-micro MS with polarity-switching (positive mode 3 kV, negative mode 2 kV), and multiple reaction monitoring mode was used to acquire data with a randomized injection order. Before, during, and after the run, quality control (QC) samples were injected. Data were processed through TargetLynx (v4.1) to identify peaks from Total Intensity Chromatograms. Peaks were then integrated, and ion counts were obtained and exported for further processing in R. Metabolites found in < 50% QC samples or those with a coefficient of variation > 30% were dropped. In addition, QC samples were used to fit a cross-validated locally estimated scatterplot smoothing (LOESS) function to each metabolite^70^. This accounted for instrumental drift and was used for ion count normalization.

### Quantification and statistical analysis

All statistical analyses were conducted using GraphPad Prism 8.0. Data represent mean ± s.e.m. unless otherwise noted and reported as biological replicates with technical replicates specified in figure legends. Unpaired two-tailed Student *t*-tests were used to determine *p*-values. Significance was defined as *p < 0.05, **p < 0.005, and ***p < 0.0005.

### Reporting summary

Further information on research design is available in the Nature Research Reporting Summary linked to this article.

### Supplementary information

Supplementary Information

Peer Review File

Reporting Summary

Description of Additional Supplementary Files

Supplementary Data 1

Supplementary Data 2

Supplementary Data 3

### Source data

Source data

## Data Availability

The data that support the findings of this study are available from the corresponding author upon reasonable request. The source data underlying Figs. 1b, d–g, 2a, b, d–f, h, 3b–e, 4a, b, e–g, 5a–d, 6a–c, e, and 7c, f–h, and Supplementary Figs. 1c, d, g–j, 2a–d, f, g, 3a, c, 4a–d, f, h, 5a–g, 6a–d, f, and 7a, b are provided as Source Data files. Metabolomics data described in Fig. 1c–e and Supplementary Fig. 1a–h are provided as Supplementary Data 1–3.
