# Supplementary material for: Targeting glutamine metabolism slows soft tissue sarcoma growth
Source: Nat Commun. 2020 Jan 24;11:498. doi: 10.1038/s41467-020-14374-1 (PMC6981153; doi:10.1038/s41467-020-14374-1)
Supplement: Supplementary file 1 — Supplementary Information [file 41467_2020_14374_MOESM1_ESM.pdf]

Supplementary Information for

Targeting Glutamine Metabolism Slows Soft Tissue Sarcoma Growth

Lee et al.

SUPPLEMENTARY FIGURES
Supplementary Figure 1

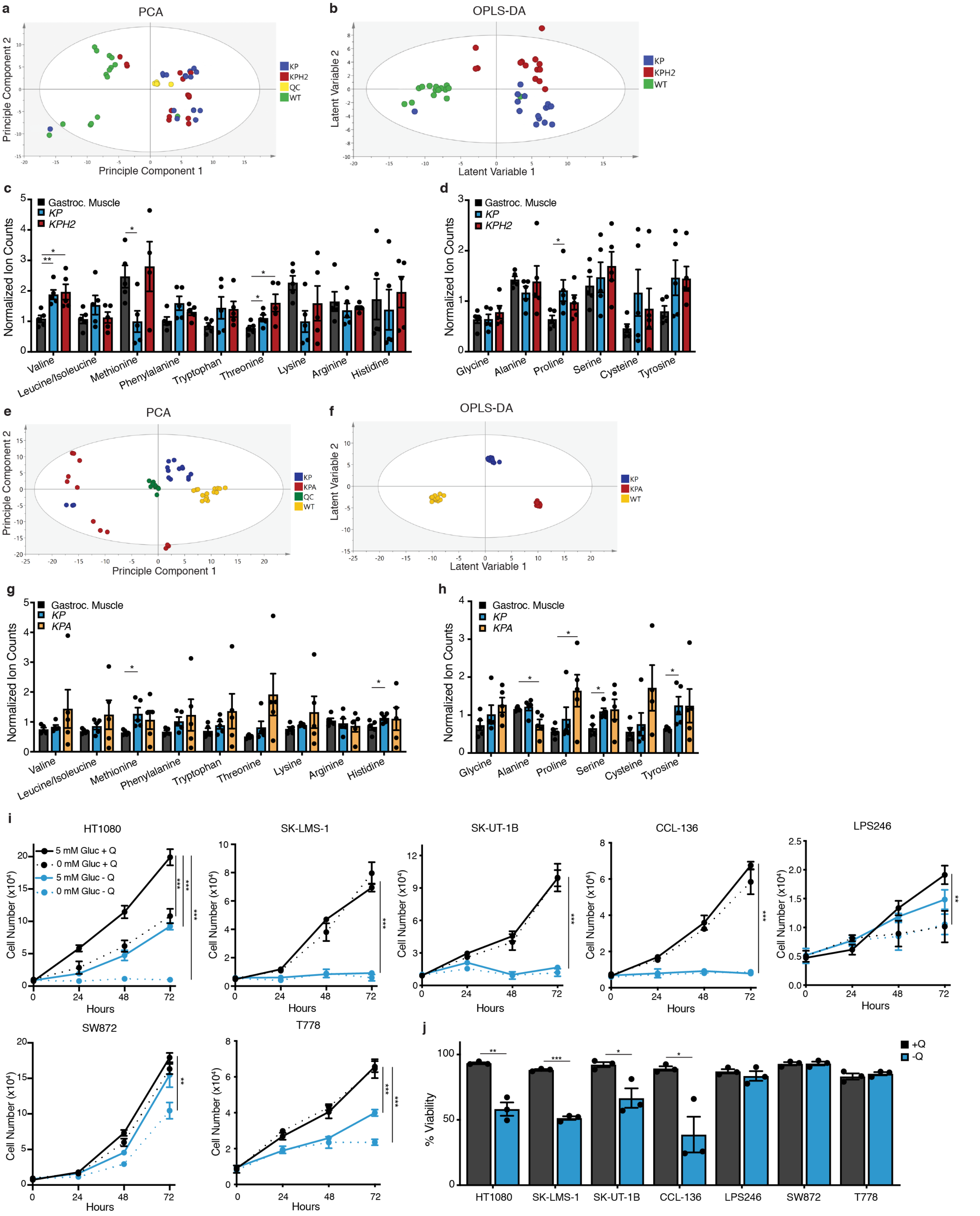

Supplementary Figure 1. UPS tumours and cells show glutamine dependency.
(A) Principle Component Analysis (PCA) plot showing the clustering of quality control (QC; yellow), gastrocnemius muscle (WT; green), *KP* (blue), and *KPH2* (red) tumours. N = 4 with 3 technical replicates.
(B) Orthogonal Projections to Latent Structures Discriminant Analysis (OPLS-DA) plot showing metabolic variations between samples from (B). N = 4 with 3 technical replicates.
(C) Normalized ion counts for essential amino acids in gastrocnemius muscle (gastroc. muscle), *KP*, and *KPH2* tumours. N = 4 with 3 technical replicates. Data represent mean ± s.d. *p<0.05, **p<0.005.
(D) Normalized ion counts for non-essential amino acids in gastroc. muscle, *KP*, and *KPH2* tumours. N = 4 with 3 technical replicates. Data represent mean ± s.d.*p<0.05.
(E) PCA plot showing the clustering of QC (green), gastrocnemius muscle (WT; yellow), *KP* (blue), and *KPA* (red) tumours. N = 4 with 3 technical replicates.
(F) OPLS-DA plot showing metabolic variations between samples from (E). N = 4 with 3 technical replicates each.
(G) Normalized ion counts for essential amino acids in gastroc. muscle, *KP*, and *KPA* tumours. N = 4 with 3 technical replicates. Data represent mean ± s.d. *p<0.05.
(H) Normalized ion counts for non-essential amino acids in gastroc. muscle, *KP*, and *KPA* tumours. N = 4 with 3 technical replicates. Data represent mean ± s.d. *p<0.05.
(I) Proliferation of human HT1080s, SK-LMS-1s, SK-UT-1Bs, CCL-136s, LPS246s (top row, left to right), SW872s, and T778s (bottom row, left to right) grown in media with or without glucose (Gluc) and/or glutamine (Q). N = 3 with 3 technical replicates. Data represent mean ± s.e.m. **p<0.005, ***p<0.0005.
(J) Viability of HT1080s, SK-LMS-1s, SK-UT-1Bs, CCL-136s, LPS246s, SW872s, and T778s grown in media with or without Q and assessed after 48 hrs. N = 3 with 2 technical replicates. Data represent mean ± s.e.m. *p<0.05, **p<0.005. ***p<0.0005.
P-values were calculated from a two-tailed Student's t-test for parts c, d, g-j. Source data are provided as a Source Data file.

Supplementary Figure 2

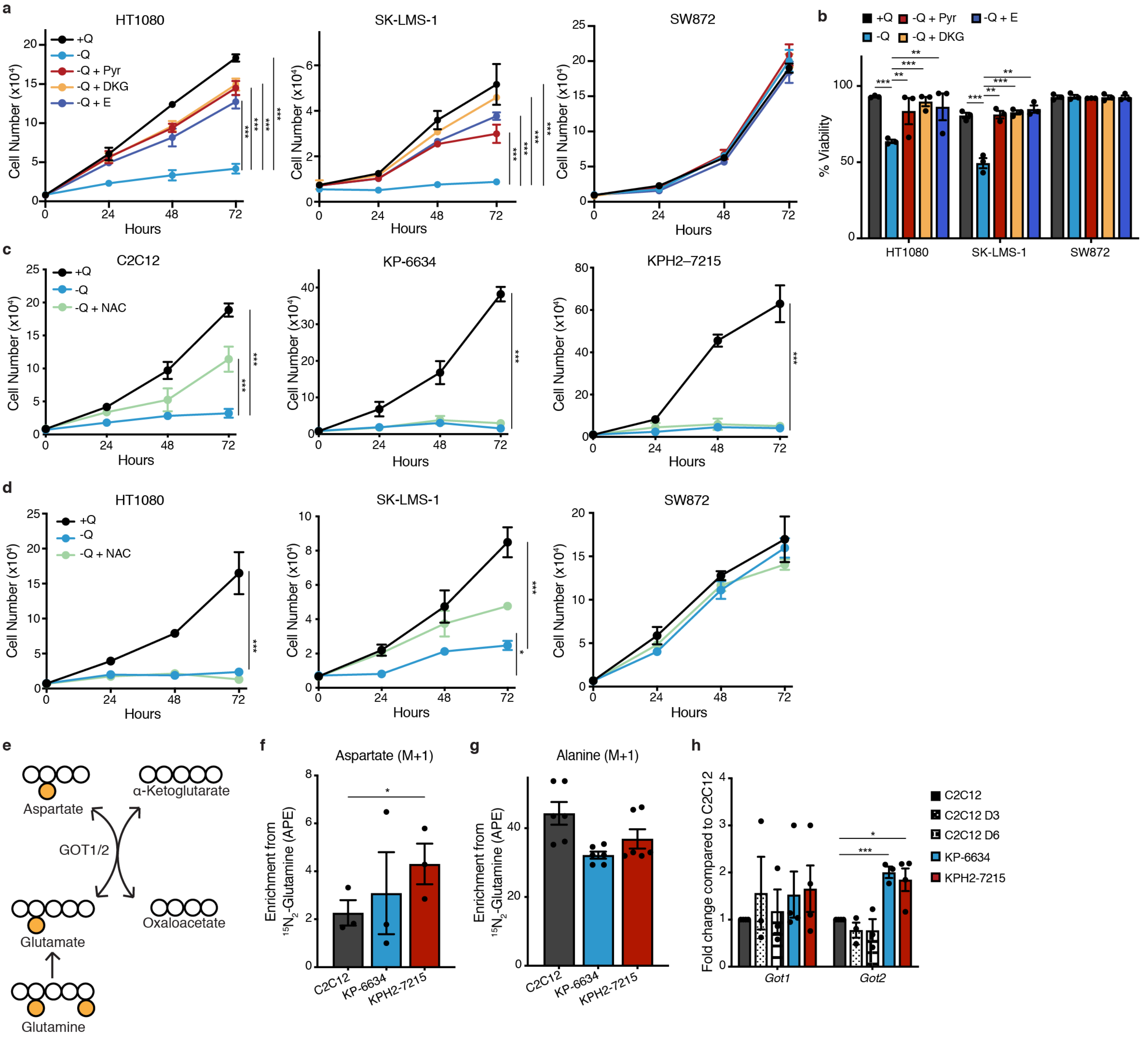

Supplementary Figure 2. UPS tumours cells maintain glutamine anaplerosis.
(A) Proliferation of HT1080s, SK-LMS-1s, and SW872s (left to right) grown in media with or without glutamine (Q), supplemented with 5 mM pyruvate (Pyr), 5 mM dimethyl 2-oxoglutarate (DKG) or 2 mM glutamate (E). N = 3 with 3 technical replicates. Data represent mean ± s.e.m. ***p<0.0005.
(B) Viability of HT1080s, SK-LMS-1s, and SW872s grown in media with or without Q, supplemented with 5 mM Pyr, 5 mM DKG or 2 mM E and assessed after 48 hrs. N = 3 with 2 technical replicates. Data represent mean ± s.e.m. **p<0.005, ***p<0.0005.
(C) Proliferation of C2C12s, KP-6634s, and KPH2-7215s (left to right) grown in media with or without Q, supplemented with 2 mM N-acetyl cysteine (NAC). N = 3 with 3 technical replicates. Data represent mean ± s.e.m. ***p<0.0005.
(D) Proliferation of HT1080s, SK-LMS-1s, and SW872s (left to right) grown in media with or without Q, supplemented with 2 mM N-acetyl cysteine (NAC). N = 3 with 3 technical replicates. Data represent mean ± s.e.m. *p<0.05, ***p<0.0005.
(E) Schematic of glutamate transamination to generate aspartate by glutamate-oxaloacetate transaminase 1/2 (GOT1/2). Filled orange circles represent ^{15}N atoms derived from $^{15}N_2$-glutamine.
(F) Mass isotopomer analysis of M+1 enrichment in aspartate, represented as atoms percent excess (APE), in C2C12s, KP-6634s, and KPH2-7215 cells cultured for 5 hrs with $^{15}N_2$-glutamine. N = 3 with 2 technical replicates. Data represent mean ± s.e.m. *p<0.05.
(G) Mass isotopomer analysis of M+1 enrichment in alanine, represented as APE, in C2C12s, KP-6634s, and KPH2-7215s cultured for 5 hrs with $^{15}N_2$-glutamine. N = 3 with 2 technical replicates. Data represent mean ± s.e.m.
(H) *Got1* and *Got2* mRNA levels in C2C12, C2C12 D3, C2C12 D6, KP-6634, and KPH2-7215 cells. N = 3 with 3 technical replicates. Data represent mean ± s.e.m. *p<0.05, ***p<0.0005.
P-values were calculated from a two-tailed Student's t-test for parts a-d, f, g. Source data are provided as a Source Data file.

Supplementary Figure 3

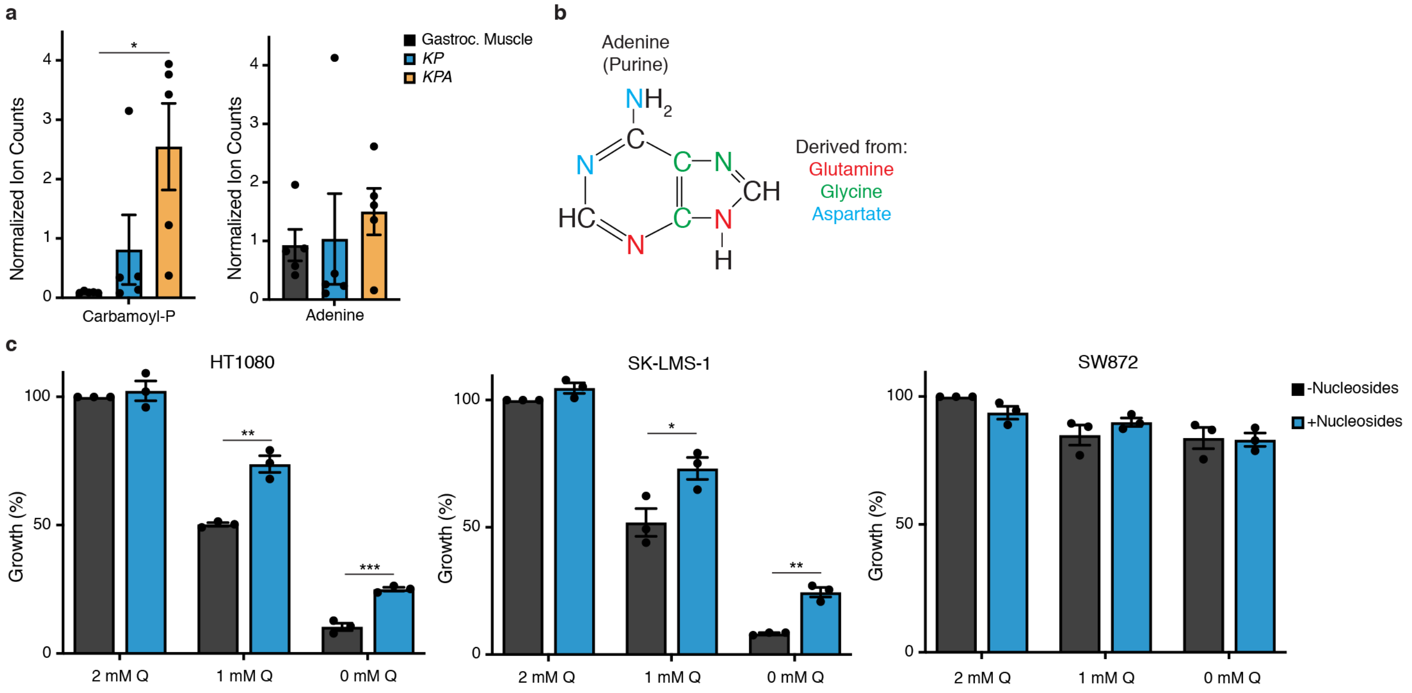

Supplementary Figure 3. Glutamine anaplerosis promotes nucleoside production in UPS tumour cells.
(A) Normalized ion counts for carbamoyl-phosphate (carbamoyl-p; left) and adenine (right) in gastrocnemius muscle (gastroc. muscle), *KP*, and *KPA* tumours. N = 4 with 3 technical replicates. Data represent mean ± s.d. *p<0.05.
(B) Diagram of the biosynthetic origins of purine ring atoms. Components derived from glutamine (red), glycine (green), and aspartate (blue) are highlighted.
(C) Growth of HT1080s, SK-LMS-1s, and SW872s (left to right) grown in media with 0 – 2 mM glutamine (Q) and supplemented with 50 µM adenosine, guanosine, cytidine and thymidine (Nucleosides) for 72 hrs. N = 3 with 3 technical replicates. Data represent mean ± s.e.m. *p<0.05, **p<0.005.
P-values were calculated from a two-tailed Student's t-test for parts a, c. Source data are provided as a Source Data file.

Supplementary Figure 4

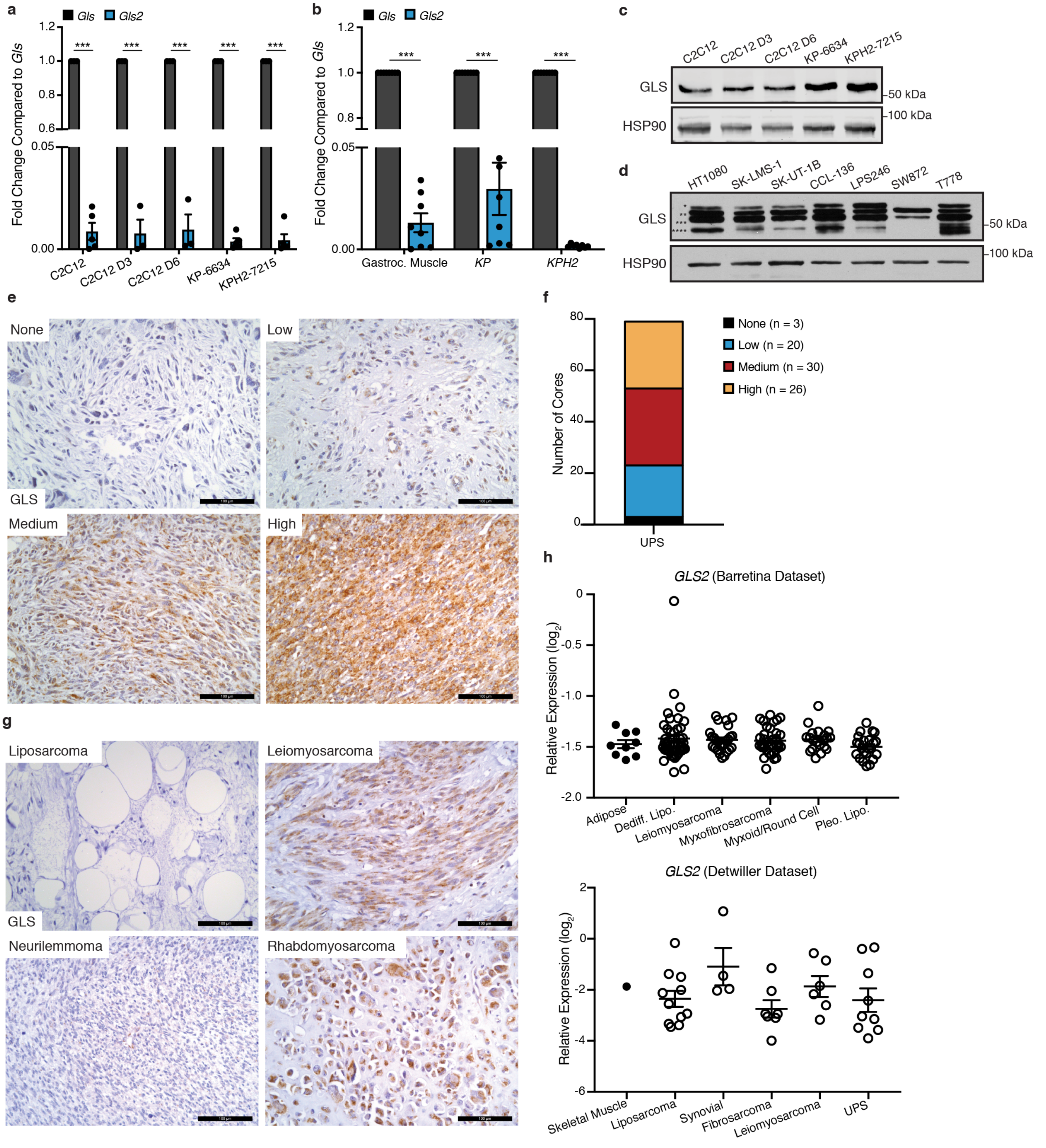

Supplementary Figure 4. Glutaminase 1 is highly expressed in UPS.
(A) *Gls* and *Gls2* mRNA levels in C2C12, C2C12 D3, C2C12 D6, KP-6634, and KPH2-7215 cells. N = 3 with 3 technical replicates. Data represent mean ± s.e.m. ***p<0.0005.
(B) *Gls* and *Gls2* mRNA levels in gastrocnemius muscle (gastroc. muscle), *KP*, and *KPH2* tumours. N = 5 with 3 technical replicates. Data represent mean ± s.e.m. ***p<0.0005.
(C) GLS protein abundance in C2C12, C2C12 D3, C2C12 D6, KP-6634, and KPH2-7215 cells.
(D) GLS protein abundance in HT1080s, SK-LMS-1s, SK-UT-1Bs, CCL-136s, LPS246s, SW872s, and T778s. *, KGA isoform; **, unspecific band; ***, GAC isoform; ****, unspecific band; as determined by manufacturer's datasheet.
(E) Representative GLS protein staining used to determine quantification scoring of GLS staining intensity (none, low, medium, or high) in an undifferentiated pleomorphic sarcoma (UPS) tissue microarray. N = 79. Scale bars = 100 μm.
(F) Quantification of GLS staining intensity in an UPS tissue microarray in (E).
(G) Representative GLS protein staining in a variety of soft tissue sarcoma (STS) subtypes from an STS tissue microarray. Scale bars = 100 μm.
(H) *GLS2* expression from Oncomine analysis of Barretina *et al.*[1] (top) and Detwiller *et al.*[2] (bottom) sarcoma patient samples data sets. Values are normalized to median-centered intensity and shown on a $\log_2$ scale. Dediff. Lipo., dedifferentiated liposarcoma; pleo. lipo., pleomorphic liposarcoma; UPS, undifferentiated pleomorphic sarcoma.
P-values were calculated from a two-tailed Student's t-test for parts a, b, h. Source data are provided as a Source Data file.

Supplementary Figure 5

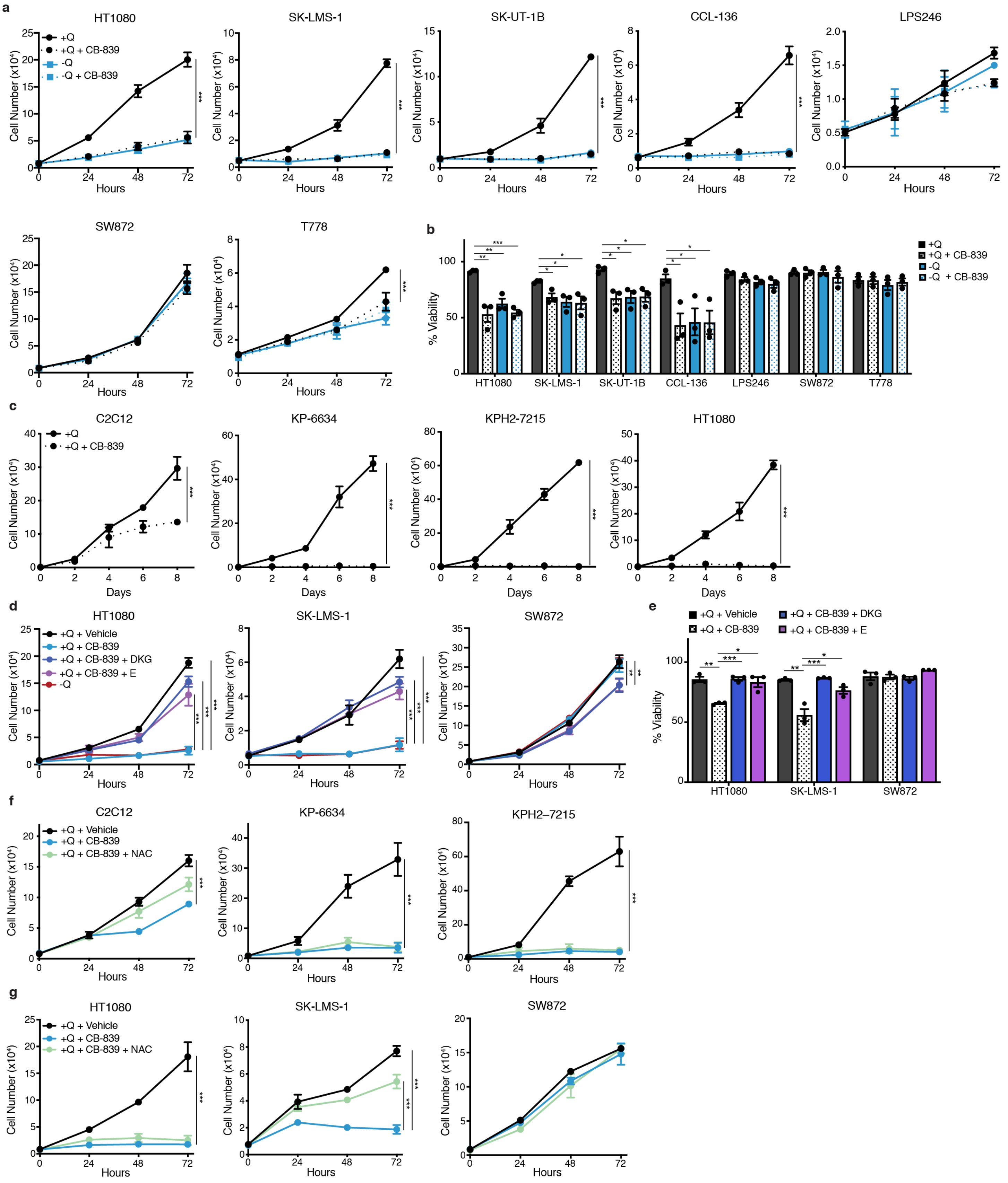

Supplementary Figure 5. Glutaminase inhibition with CB-839 targets UPS cells.
(A) Proliferation of HT1080s, SK-LMS-1s, SK-UT-1Bs, CCL-136s, LPS246s (top row, left to right), SW872s, and T778s (bottom row, left to right) grown in media with or without glutamine (Q) and treated with 1 μM CB-839. N = 3 with 3 technical replicates. Data represent mean ± s.e.m. ***p<0.0005.
(B) Viability of HT1080s, SK-LMS-1s, SK-UT-1Bs, CCL-136s, LPS246s, SW872s, and T778s grown in media with or without Q, treated with 1 μM CB-839 and assessed after 48 hrs. N = 3 with 2 technical replicates. Data represent mean ± s.e.m. *p<0.05, **p< 0.005, ***p<0.0005.
(C) Proliferation of C2C12s, KP-6634s, KPH2-7215s, and HT1080s (left to right) grown in media with Q and treated with 1 μM CB-839 for 8 days. N = 3 with 3 technical replicates. Data represent mean ± s.e.m. ***p<0.0005.
(D) Proliferation of HT1080s, SK-LMS-1s, and SW872s (left to right) grown in media with or without Q, treated with 1 μM CB-839, and 5 mM dimethyl 2-oxoglutarate (DKG) or 2 mM glutamate (E). N = 3 with 3 technical replicates. Data represent mean ± s.e.m. **p<0.005, ***p<0.0005.
(E) Viability of HT1080s, SK-LMS-1s, and SW872s grown in media with or without Q, treated with 1 μM CB-839, and 5 mM DKG or 2 mM E, and assessed after 48 hrs. N = 3 with 2 technical replicates. Data represent mean ± s.e.m. *p<0.05, **p<0.005, ***p<0.0005.
(F) Proliferation of C2C12s, KP-6634s, and KPH2-7215s (left to right) grown in media with Q, treated with 1 μM CB-839, and 2 mM N-acetyl cysteine (NAC). N = 3 with 3 technical replicates. Data represent mean ± s.e.m. ***p<0.0005.
(G) Proliferation of HT1080s, SK-LMS-1s, and SW872s (left to right) grown in media with Q, treated with 1 μM CB-839, and 2 mM NAC. N = 3 with 3 technical replicates. Data represent mean ± s.e.m. ***p<0.0005.
P-values were calculated from a two-tailed Student's t-test for parts a-g. Source data are provided as a Source Data file.

Supplementary Figure 6

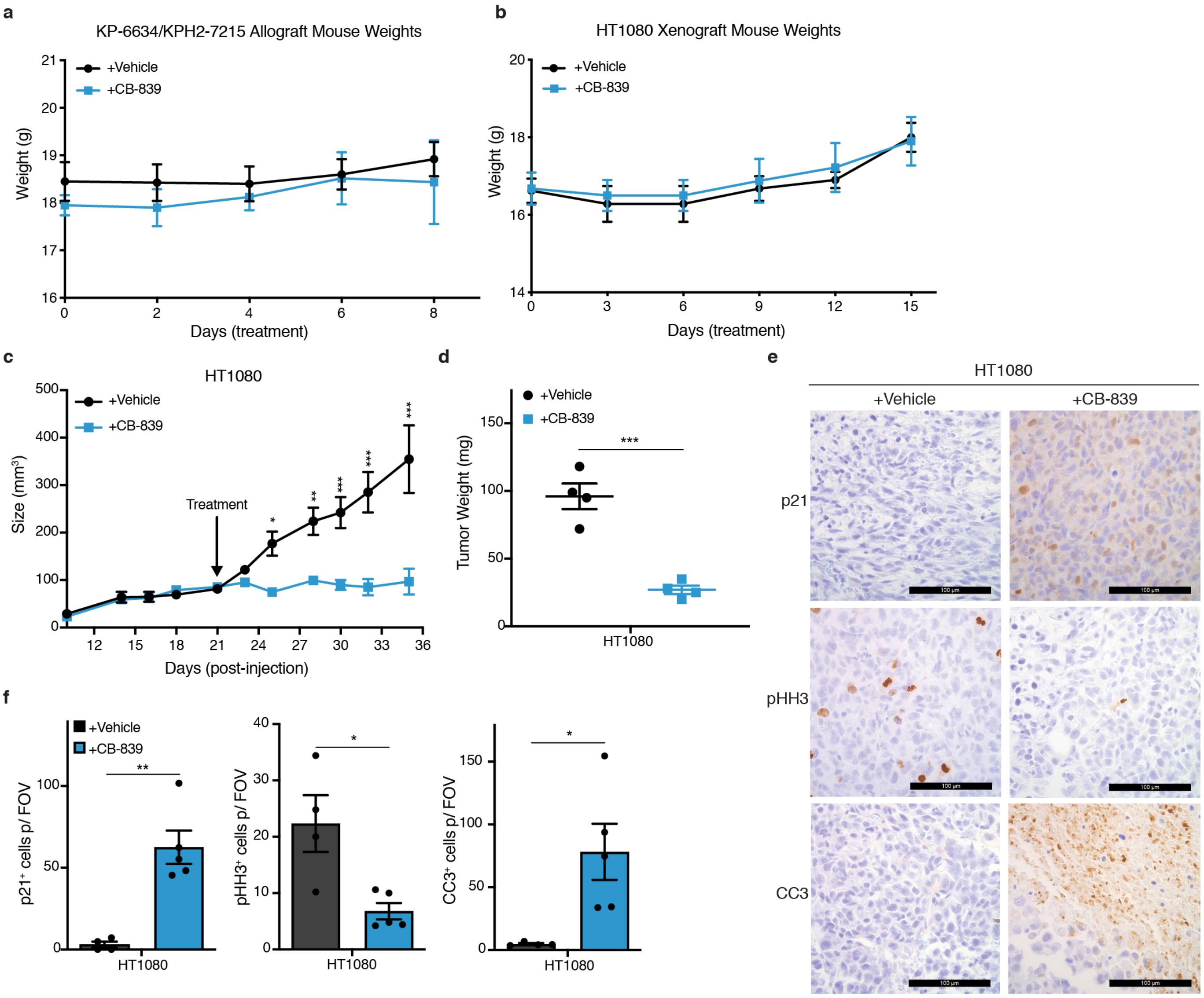

Supplementary Figure 6. Glutaminase inhibition effectively blunts UPS allograft and xenograft growth.

(A) Mouse weights from vehicle- (n = 4) and CB-839-treated (n = 4) KP-6634/KPH2-7215 allograft-bearing mice following 8 days of treatment. Data represent mean ± s.d.

(B) Mouse weights from vehicle- (n = 4) and CB-839-treated (n = 4) HT1080 xenograft-bearing mice following 15 days of treatment. Data represent mean ± s.d.

(C) Tumour size of subcutaneous HT1080 xenografts in vehicle- or CB-839-treated mice (n = 4). Mice were treated once tumours reached ~100 mm^3 at ~21 days post-injection. Data represent mean ± s.d. *p<0.05, **p<0.005, ***p<0.0005.

(D) Tumour weights from vehicle- and CB-839-treated HT1080 xenograft mice (n = 4). Data represent mean ± s.d. ***p<0.0005.

(E) Representative immunohistochemistry staining for p21 (top row), phospho-histone H3 (middle row; pHH3), and cleaved-caspase 3 (bottom row; CC3) in HT1080 xenograft vehicle- (n = 4) or CB-839-treated (n = 4) animals. Scale bars = 100 μm.

(F) Quantification of $p21^+$, $pHH3^+$, and $CC3^+$ (left to right) cells per field of vision (FOV) in vehicle- and CB-839-treated HT1080 xenograft animals. N = 4 with 5 images taken per section. Data represent mean ± s.e.m. *p<0.05, **p<0.005. *P*-values were calculated from a two-tailed Student's t-test for parts a-d, f. Source data are provided as a Source Data file.

Supplementary Figure 7

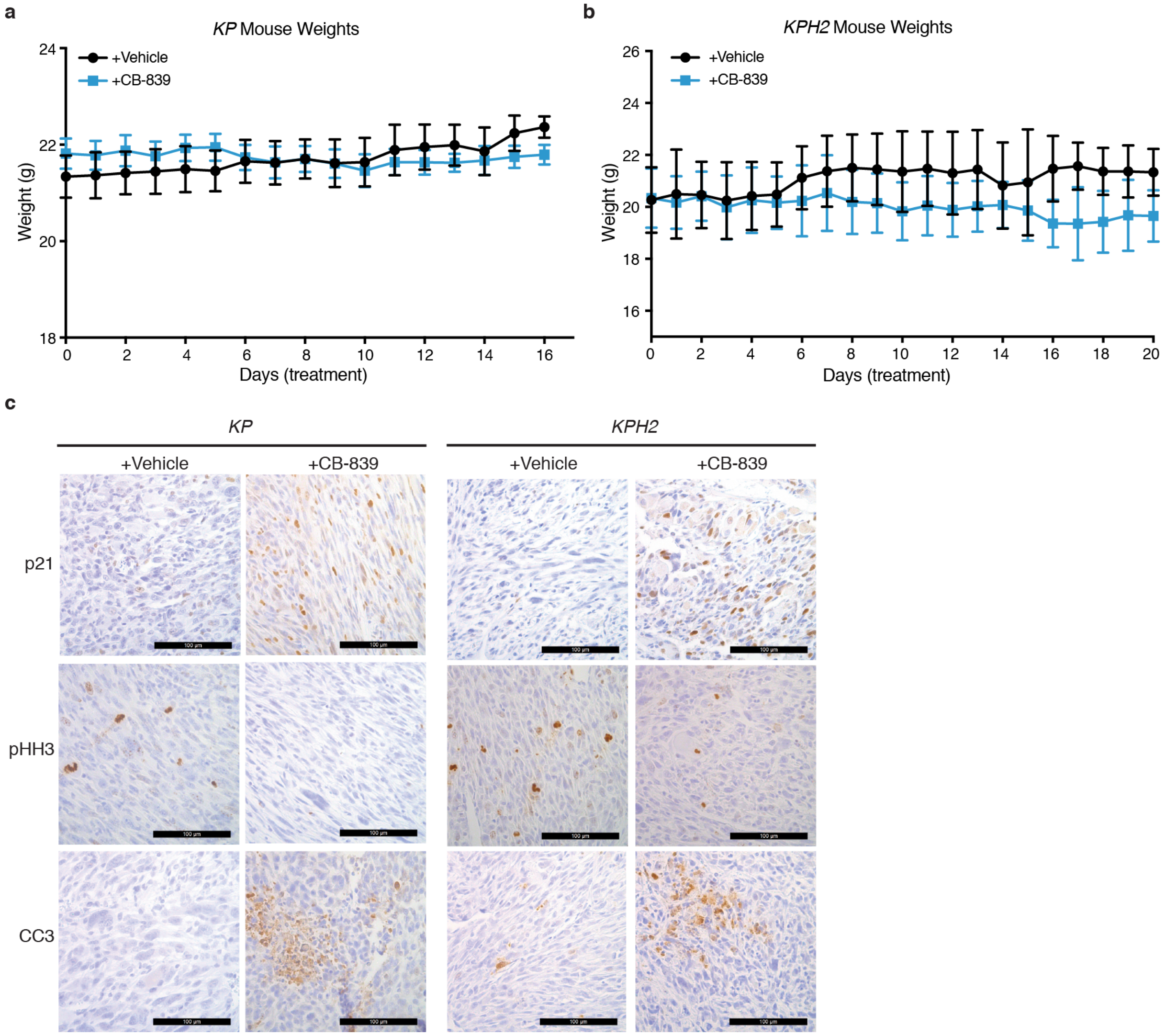

Supplementary Figure 7. CB-839 is effective in an autochthonous UPS model.
(A) Mouse weights from vehicle- (n = 8) and CB-839-treated (n = 10) *KP* mice following up to 16 days of treatment. Data represent mean ± s.d.
(B) Mouse weights from vehicle- (n = 9) and CB-839-treated (n = 10) *KPH2* mice following up to 21 days of treatment. Data represent mean ± s.d.
(C) Representative immunohistochemistry staining for p21 (top), phospho-histone H3 (middle; pHH3), and cleaved-caspase 3 (bottom; CC3) in vehicle- and CB-839-treated *KP* (vehicle n = 8; CB-839 n = 10) and *KPH2* (vehicle n = 9; CB-839 n = 10) animals. Scale bars = 100 μm.
P-values were calculated from a two-tailed Student's t-test for parts a, b. Source data are provided as a Source Data file.

Supplementary Figure 8

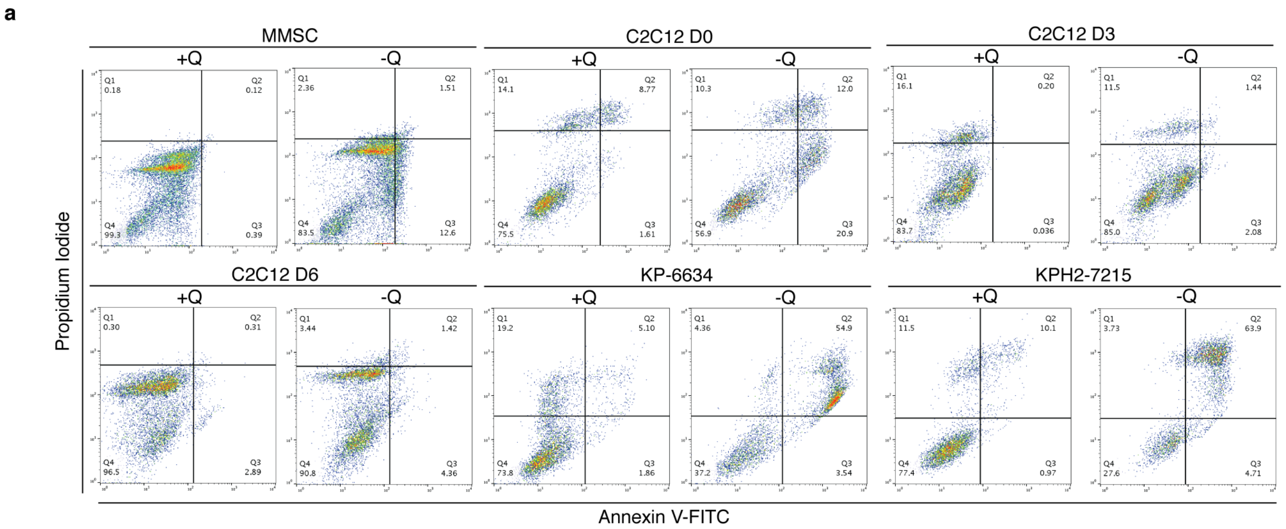

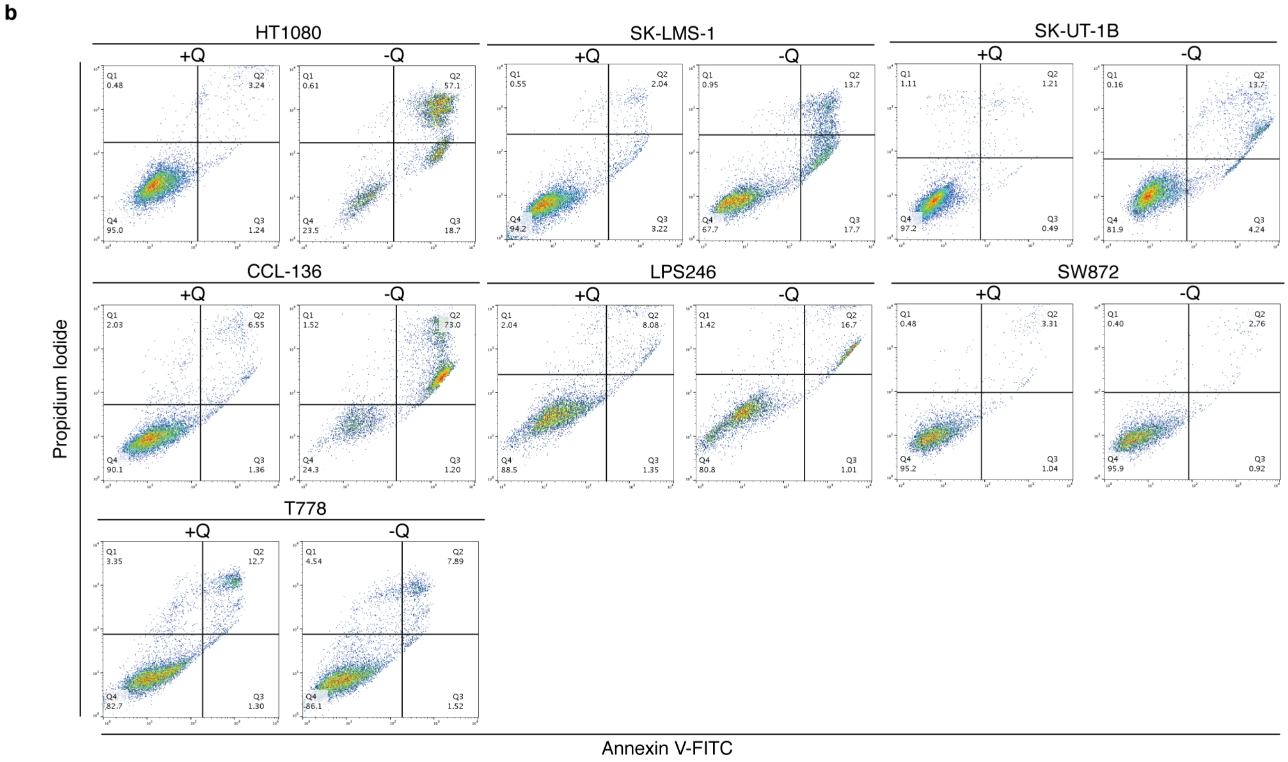

Supplementary Figure 8. Gating strategy for flow cytometry data presented as histograms in the manuscript.
(A) Flow plots exemplifying the gating strategy for Fig. 1G using Annexin VI-FITC/Propidium Iodide.
(B) Flow plots exemplifying the gating strategy for Supplementary Fig. 1J using Annexin VI-FITC/Propidium Iodide.

SUPPLEMENTARY REFERENCES

1. Barretina, J. *et al.* Subtype-specific genomic alterations define new targets for soft-tissue sarcoma therapy. *Nat. Genet.* **42**, 715–21 (2010).
2. Detwiller, K. Y. *et al.* Analysis of Hypoxia-Related Gene Expression in Sarcomas and Effect of Hypoxia on RNA Interference of Vascular Endothelial Cell Growth Factor A. *Cancer Res.* **65**, 5881–5889 (2005).
