# Supplementary material for: Targeting glutamine metabolism slows soft tissue sarcoma growth
Source: Nat Commun. 2020 Jan 24;11:498. doi: 10.1038/s41467-020-14374-1 (PMC6981153; doi:10.1038/s41467-020-14374-1)
Supplement: Supplementary file 3 — Reporting Summary [file 41467_2020_14374_MOESM3_ESM.pdf]

nature research

Corresponding author(s): M. Celeste Simon

Last updated by author(s): Dec 16, 2019

Reporting Summary

Nature Research wishes to improve the reproducibility of the work that we publish. This form provides structure for consistency and transparency in reporting. For further information on Nature Research policies, see Authors & Referees and the Editorial Policy Checklist.

Statistics

For all statistical analyses, confirm that the following items are present in the figure legend, table legend, main text, or Methods section.

n/a	Confirmed	
☐	☒	The exact sample size (*n*) for each experimental group/condition, given as a discrete number and unit of measurement
☐	☒	A statement on whether measurements were taken from distinct samples or whether the same sample was measured repeatedly
☐	☒	The statistical test(s) used AND whether they are one- or two-sided<br>*Only common tests should be described solely by name; describe more complex techniques in the Methods section.*
☐	☒	A description of all covariates tested
☐	☒	A description of any assumptions or corrections, such as tests of normality and adjustment for multiple comparisons
☐	☒	A full description of the statistical parameters including central tendency (e.g. means) or other basic estimates (e.g. regression coefficient) AND variation (e.g. standard deviation) or associated estimates of uncertainty (e.g. confidence intervals)
☐	☒	For null hypothesis testing, the test statistic (e.g. F, t, r) with confidence intervals, effect sizes, degrees of freedom and P value noted<br>*Give P values as exact values whenever suitable.*
☒	☐	For Bayesian analysis, information on the choice of priors and Markov chain Monte Carlo settings
☒	☐	For hierarchical and complex designs, identification of the appropriate level for tests and full reporting of outcomes
☒	☐	Estimates of effect sizes (e.g. Cohen's *d*, Pearson's *r*), indicating how they were calculated

Our web collection on statistics for biologists contains articles on many of the points above.

Software and code

Policy information about availability of computer code

Data collection	Software was not used for data collection.
Data analysis	Tumour volumes from CT scans were quantified using ITK-SNAP 3.6.0. Metabolomic data was processed and integrated using Waters TargetLynx (v4.1) and further processed in R. Flow cytometry data was analyzed on FlowJo v.10.4.1. Statistical analyses were conducted using GraphPad Prism 8.0.

For manuscripts utilizing custom algorithms or software that are central to the research but not yet described in published literature, software must be made available to editors/reviewers. We strongly encourage code deposition in a community repository (e.g. GitHub). See the Nature Research guidelines for submitting code & software for further information.

Data

Policy information about availability of data

All manuscripts must include a data availability statement. This statement should provide the following information, where applicable:

- Accession codes, unique identifiers, or web links for publicly available datasets
- A list of figures that have associated raw data
- A description of any restrictions on data availability

The data that support the findings of this study are available from the corresponding author upon reasonable request.

Field-specific reporting

Please select the one below that is the best fit for your research. If you are not sure, read the appropriate sections before making your selection.

☒ Life sciences ☐ Behavioural & social sciences ☐ Ecological, evolutionary & environmental sciences

For a reference copy of the document with all sections, see nature.com/documents/nr-reporting-summary-flat.pdf

Life sciences study design

All studies must disclose on these points even when the disclosure is negative.

Sample size	In vitro assays: Sample size calculations were not performed. Data reported as minimum of 3 biological replicates with minimum of 2 technical replicates. This was enough to achieve significance with student's t test.<br>Animal studies: Sample size for each experiment was estimated using the formula n=((zα/2 σ)/E)2, with α=0.05, and σ and E based off initial KP experiments with vehicle or CB-839 treatment.
Data exclusions	No data were excluded from analyses.
Replication	Experimental replication was successful. Data reported as minimum of 3 biological replicates with minimum of 2 technical replicates.
Randomization	In vitro assays: Samples were not randomized. Barring treatment, covariates remained the same across sample groups.<br>Animal studies: Following AdCre injection or subcutaneous injection of cells (allograft/xenograft studies), animals were randomized into groups for vehicle or drug treatment.
Blinding	Animal studies: Researchers were not blinded to the experimental groups during in vivo treatments to ensure correct groups were treated.<br>In vitro assays: Researchers were not blinded to the experimental groups to ensure correct groups were treated. Certain methods removed bias, such as flow cytometry, qPCR, and immunoblotting, as samples were internally controlled.<br>CT Imaging and Analysis: Researchers were blinded to experimental groups to remove bias. Researcher performing treatment was not the same as researchers performing imaging and analysis.<br>Immunohistochemistry: Researchers were blinded to experimental groups/slides to remove bias on protein expression.

Reporting for specific materials, systems and methods

We require information from authors about some types of materials, experimental systems and methods used in many studies. Here, indicate whether each material, system or method listed is relevant to your study. If you are not sure if a list item applies to your research, read the appropriate section before selecting a response.

Materials & experimental systems

n/a	Involved in the study
☐	☒ Antibodies
☐	☒ Eukaryotic cell lines
☒	☐ Palaeontology
☐	☒ Animals and other organisms
☒	☐ Human research participants
☒	☐ Clinical data

Methods

n/a	Involved in the study
☒	☐ ChIP-seq
☐	☒ Flow cytometry
☒	☐ MRI-based neuroimaging

Antibodies

Antibodies used	GLS (Abcam; Cat. #ab156876), HSP90 (Cell Signaling; Cat. #4874), cleaved-caspase 3 (Cell Signaling; Cat. #9661), p21 (Abcam; Cat. #ab188224), phospho-Histone H3 (Cell Signaling; Cat #3377).
Validation	GLS antibody is knockout validated by the manufacturer. HSP90, cleaved-caspase 3, and phospho-histone H3 antibodies are highly cited on the manufacturer's (Cell Signaling) page. p21 antibody is verified by manufacturer (Abcam) using NIH/3T3 cells untreated and treated with 1µM staurosporine for 2 hrs.

Eukaryotic cell lines

Policy information about cell lines

Cell line source(s)	HT1080, SK-LMS-1, SK-UT-1B, CCL-136, SW872, 94T778 (T778), and C2C12 cells were purchased from ATCC. LPS246 were provided by Dr. Dina Lev (MD Anderson Cancer Center). KP-6634s and KPH2-7215s were derived from UPS mouse tumours from previous study (Nakazawa et al, 2016). Murine mesenchymal stem cells (MMSCs) were purchased from Cyagen.

Authentication	Cell lines were previously authenticated, but not for this study.
Mycoplasma contamination	All cells are routinely confirmed as Mycoplasma negative (MycoAlert; tested every 3 months).
Commonly misidentified lines (See ICLAC register)	None of the cell lines used in this study were on the ICLAC register.

Animals and other organisms

Policy information about studies involving animals; ARRIVE guidelines recommended for reporting animal research

Laboratory animals	Tumours were generated in 8 weeks or older male and females LSL-KrasG12D/+;Trp53fl/fl (KP), LSL-KrasG12D/+;Trp53fl/fl;Epas1fl/fl (KPH2), and LSL-KrasG12D/+;Trp53fl/fl;Arntfl/fl (KPA). For xenografts and allografts, 8 week old female Balb/c nu/nu mice were used (Charles River Laboratories).
Wild animals	N/A
Field-collected samples	N/A
Ethics oversight	Animal wellbeing was monitored by certified veterinary staff. All mouse experiments were performed according to National Institutes of Health guidelines and approved by the University of Pennsylvania Institutional Animal Care and Use Committee.

Note that full information on the approval of the study protocol must also be provided in the manuscript.

Flow Cytometry

Plots

Confirm that:

- [x] The axis labels state the marker and fluorochrome used (e.g. CD4-FITC).
- [x] The axis scales are clearly visible. Include numbers along axes only for bottom left plot of group (a 'group' is an analysis of identical markers).
- [x] All plots are contour plots with outliers or pseudocolor plots.
- [x] A numerical value for number of cells or percentage (with statistics) is provided.

Methodology

Sample preparation	Cells (1x10^5 p/mL) were seeded in maintenance media in 6-well plates and allowed to adhere overnight before receiving assay media. After 48 hrs, cell viability was assessed by FITC-Annexin V/PI Kit (BD Biosciences; Cat. #556547) according to manufacturer's instructions.
Instrument	Flow cytometry was performed using a BD FACSCalibur.
Software	Flow cytometry data was analyzed on FlowJo v.10.4.1. Data presented as histograms.
Cell population abundance	For each experiment, a total of 10,000 cells were counted.
Gating strategy	Cell populations were first gated by FSC-H/SSC-A. Cell viability was then assessed by Annexin V-FITC/PI staining using FL1-A/FL3-A. Positive population (viable) cells were determined as double negative (control group). Negative population (non-viable) cells were determined as single- or double-positive cells. Examples for gating strategy are found in Supplementary Fig. 8.

- [x] Tick this box to confirm that a figure exemplifying the gating strategy is provided in the Supplementary Information.
