# Supplementary material for: Targeting glutamine metabolism slows soft tissue sarcoma growth
Source: Nat Commun. 2020 Jan 24;11:498. doi: 10.1038/s41467-020-14374-1 (PMC6981153; doi:10.1038/s41467-020-14374-1)
Supplement: Supplementary file 4 — Description of Additional Supplementary Files [file 41467_2020_14374_MOESM4_ESM.docx]

**Description of Additional Supplementary Files**

File name: Supplementary Data 1
Description: Metabolomics data of gastrocnemius muscle (gastroc. muscle), KP, and KPH2 samples represented in Fig. 1C-D, Fig. 3B, and Supplementary Fig. 1A-D. Data presented of one in vivo experiment (5 biological samples with 3 technical replicates each). NA indicates undetected measurements.

File name: Supplementary Data 2
Description: Metaboanalyst integrated pathway analysis of gastrocnemius muscle and KPH2 tumour metabolomics (Supplementary Data 1). Enrichment of highlighted pathways presented in Fig. 1C.

File name: Supplementary Data 3
Description: Metabolomics data of gastrocnemius muscle (gastroc. muscle), KP, and KPA samples represented in Fig. 1E, Supplementary Fig. 1E-H, and Supplementary Fig. 3A. Data presented of one in vivo experiment (5 biological samples with 3 technical replicates each). NA indicates undetected measurements.
