# Supplementary material for: Targeting glutamine metabolism slows soft tissue sarcoma growth
Source: Nat Commun. 2020 Jan 24;11:498. doi: 10.1038/s41467-020-14374-1 (PMC6981153; doi:10.1038/s41467-020-14374-1)
Supplement: Supplementary file 2 — Peer Review File [file 41467_2020_14374_MOESM2_ESM.pdf]

Reviewers' comments:

Reviewer #1 (Remarks to the Author); expert in metabolism:

The manuscript by Lee and colleagues investigates the metabolic features of soft tissue sarcomas (STS), combining mouse models based on expression of oncogenic Kras and p53 loss and a panel of human-derived cancer lines. Both in vitro and in vivo, STS tumors display features of altered glutamine metabolism. The authors report very interesting differences in expression of glutaminase across multiple STS subtypes and show that GLS expression correlates with sensitivity to glutamine withdrawal and glutaminase inhibition with the agent CB-839. While several previous studies have shown mixed efficacy of CB-839 in vivo—including in tumors with similar genetic makeup—this manuscript demonstrates promising anti-tumor properties of CB-839 in both grafting and genetically engineered mouse models of STS. Beyond revealing the metabolic signatures of a poorly studied cancer, this manuscript presents exciting data demonstrating the diversity of metabolic strategies used to support tumor growth in a tissue-specific fashion and identifies potential therapeutic strategies for targeting the metabolic phenotype of soft tissue sarcomas. This manuscript will likely be of broad interest to researchers in cancer metabolism and tumor biology, and I recommend it for publication in Nature Communications. I have only a few minor suggestions for improvement:

1. The glutamine labeling to aspartate in Fig. 2h appears quite low, especially relative to glutamine labeling to alanine. Could the authors comment on this discrepancy (is there aspartate in the medium?), or put the numbers in context?
2. In Figs. 2d,e, the authors note that the contribution of glutamine to the TCA cycle (as demonstrated by citrate) is similar, but the contribution to aspartate is increased. Because aspartate is generated from glutamine carbons via the TCA cycle, presumably glutamine contributes more to at least a portion of the TCA cycle in STS lines. Could the authors clarify this statement or include more TCA cycle metabolites in their analysis?
3. The methods note that labeling is done with DMEM (and growth curves with 'media based on DMEM'), but DMEM traditionally contains large amounts of glutamine. Could the authors clarify whether these experiments were done in glucose/glutamine-free DMEM supplemented with labeled/unlabeled versions?
4. The authors show levels of certain metabolites for some tumors and not others. I appreciate that not all data will be equivalently robust across distinct experiments, but could the authors provide a supplementary table with all of the acquired metabolomics data in which they are confident? This paper represents an important resource, and such a table will be of interest to readers in the field

Reviewer #2 (Remarks to the Author); expert in sarcoma:

In the manuscript titled "Enhanced Glutamine Metabolism Promotes Soft Tissue Sarcoma Progression" Leet et al show that murine UPS and human fibrosarcoma and leiomyosarcoma cell proliferation depends on glutamine derived glutamate to support carbon metabolism, aspartate production, and nucleotide biosynthesis. Importantly this dependency can be targeted therapeutically with the glutaminase inhibitor CB-839, currently in clinical trials for other tumor types. While previous studies in PDAC and lung cancer human lines and mouse models have shown that disparate results in regard to CB-839 sensitivity and glutamine dependence in vitro and in vivo, this study shows a promising effect of GLS inhibition in some sarcoma sub-types in vitro and in vivo in xenografts and in an autochthonous mouse model of UPS.

The manuscript is well written the study is timely and relevant for sarcoma and cancer metabolism research. The study includes both in vitro and in vivo analyses and characterization of mechanisms that underlie dependence on glutamine metabolism in murine and human sarcoma lines. Importantly,

the authors show that inhibiting GLS using CB-839 has anti-tumor activity in xenografts but also in an autochthonous mouse model of UPS which should be a better predictor of cancer metabolic dependencies when compared with subcutaneously transplanted tumors. The study is well performed and it is relevant since it identifies a potential new treatment, that could be used in combination with other current drugs for the treatment of some sub-types of soft-tissue sarcoma, a disease that remains extremely challenging to treat.

Some points should be address and clarified to improve the quality of this study:

Major Points

1. As sarcomas are an extremely heterogenous group of tumors understanding which sarcoma sub-types could benefit from GLS inhibition for treatment is important. Based on GLS levels in patient samples and analysis of three human cell lines (2 sensitive: fibrosarcoma, leiomyosarcoma; 1 resistant: liposarcoma), the authors conclude that sub-types such as UPS and leiomyosarcoma may be sensitive to GLS inhibition while "liposarcomas may rely on other metabolic pathways for their energetic and anabolic purposes".
However, I think this is the weakest aspect of this study for the following reasons:

- The authors use GLS levels (mRNA and protein), to predict which sarcoma sub-types would, in principal be sensitive to GLS inhibition. However, are GLS levels a good predictor of CB-839 sensitivity? Although it is reasonable to target GLS in GLS-high tumors, it does not appear to be a consensus in the literature for biomarkers that could predict sensitivity to CB-839. See for example (PMID: 31088535), in which CB-839 resistant breast cancer lines and xenografts exhibit high GLS mRNA and protein levels. This caveat should be discussed.

- The number of sarcoma types and human lines tested for glutamine starvation and CB-839 sensitivity is too low to accurately predict sensitive sarcoma sub-types or correlate sensitivity with GLS levels. Only 3 sarcoma human cell lines were analyzed, all from different subtypes. The one 1 resistant line (liposarcoma) leads the author to suggest liposarcoma may be an exception in regards to glutamine dependence, but one single cell line may just reflect variability within sub-types. Additionally, to say in the abstract that "Multiple STS subtypes expressing elevated glutaminase (GLS) levels are highly sensitive to glutamine starvation" does not fully reflect what was tested. The same laboratory has previously used another human liposarcoma cell line in their studies (LPS246, is this line one resistant too?), and at least one more leiomyosarcoma cell line available at ATCC. Those, and if possible additional ones, should be tested for glutamine starvation and CB-839 treatment in vitro (alongside with GLS protein levels).

Given the challenges in studying this disease (e.g. very few human cell lines and available mouse models) a comprehensive analysis may be particularly hard to accomplish. If there is variability across the few lines available, the authors should simply acknowledge that more studies in additional sarcoma models will be required to more comprehensively understand these differences.

2. One of the most interesting results in this study is that mouse models harboring similar genetic alterations (KP mice, Mutant Kras and Trp53 – deleted tumors) show different sensitivity to CB-839, suggesting that "tumour microenvironment, surrounding tissue, and cell of origin influence metabolic responses in these models". Nevertheless, it role for KRAS in re-wiring metabolism in PDAC has been described. Would it be possible that sarcomas with RAS mutations show greater dependency on glutamine metabolism (which could also reflect different GLS levels across sub-types)? Do the 2 sensitive human cell lines harbor Ras mutations? There are other sarcoma datasets available that provide both mutational analysis and mRNA expression analysis (TCGA sarcoma data set).

3. In PDAC, the ineffective results of CB-839 in vivo were associated with highly adaptive metabolic networks in these cells following sustained exposure of the GLS inhibitor (also observed in vitro). It

would be of interest to performed long-term proliferation assays in vitro with CB-839 (longer than 72h) in the sarcoma lines to determine if they remain sensitive or are equally able to re-wire metabolic dependencies which could also inform about the requirement for combination therapies.

Minor points:

- The authors re-use images from their previous publication in Nature communications (reference 41) with Figure S1A and S1F here corresponding to Figure 1B and 1D in ref 41. While the authors clearly state this model was previously described and in the figure legends that the images were adapted from the previous publication, it seems unnecessary to re-include the same images. Furthermore, while the representative tumors images and the same between studies, the graphs depicting tumor weight suggest they correspond to different cohorts (please see Fig1B in this study and Fig 1B in ref. 41).

- Page 11: "However, in marked contrast to previous in vivo studies, CB-839 administration to KP and KPH2 animals substantially inhibited tumour growth, as calculated from the difference in the muscular compartment of tumor-bearing limbs (red) relative to control limbs (green) (Fig. 7A-C; 7D-F)." Which in vivo studies are the authors referring here? There is no reference for this statement and is unclear whether the authors are refereeing to studies using sarcoma lines or other types of tumors (eg. PDAC).

- How did the authors choose a dose of 2uM for CB-839 for all lines analyzed? Were IC50 values determined, or it was based on previous studies.

- In the discussion: "Although human STSs show significant heterogeneity of GLS expression (Supplementary Fig. 4D-E), we noted a striking enrichment in UPS (Fig. 4F-H)"
There is currently no Figure 4H (both graphs are depicted as Figure 4F).

- In some human cancer tissues, increased levels of GLS1 are associated with a higher disease stage and poor prognosis PMID:25844758. Did the authors see a similar trend in samples analyzed in Fig 4E (if clinical data is available).

Reviewers' Comments:

<u>**Reviewer #1 (Remarks to the Author); expert in metabolism:**</u>

1. The glutamine labeling to aspartate in Fig. 2h appears quite low, especially relative to glutamine labeling to alanine. Could the authors comment on this discrepancy (is there aspartate in the medium?), or put the numbers in context?

The labelling experiments the reviewer refers to use ^{15}N-labeled glutamine in media lacking aspartate. While both aspartate and alanine derive their nitrogen from glutamine/glutamate through transamination, they rely on separate precursors: aspartate on oxaloacetate and alanine on pyruvate. Since these labelling experiments were not performed under glucose-free conditions, it is likely that high enrichment in alanine is due to available pyruvate pools which would drive alanine production. This has now been clarified and highlighted in the main text (**see p. 8, first paragraph**).

2. In Figs. 2d, e, the authors note that the contribution of glutamine to the TCA cycle (as demonstrated by citrate) is similar, but the contribution to aspartate is increased. Because aspartate is generated from glutamine carbons via the TCA cycle, presumably glutamine contributes more to at least a portion of the TCA cycle in STS lines. Could the authors clarify this statement or include more TCA cycle metabolites in their analysis?

Due to the transamination reaction, enrichment of aspartate from U-^{13}C-glutamine can be from labelled oxaloacetate as well as labelled glutamate. This may be why enrichment in aspartate from U-^{13}C-glutamine is higher in the sarcoma lines, despite no changes in TCA cycle intermediates. Furthermore, it may be difficult to compare the enrichment of aspartate to TCA cycle metabolites since aspartate in this case is an “end-product”, while the TCA cycle intermediates continue to cycle (**see Supplementary Fig. 2E**). Previously, we only showed enrichment into citrate and have now included other TCA cycle metabolites as the reviewer has suggested (**see Fig. 2D; p. 7, fourth paragraph**).

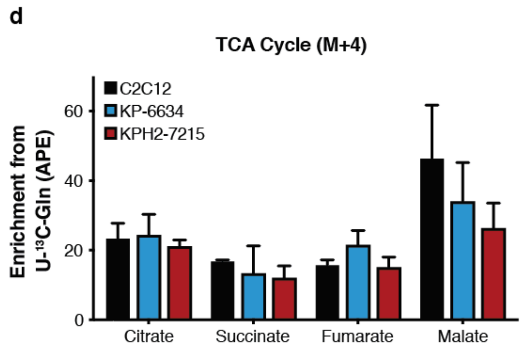

Fig. 2: (D) Mass isotopomer analysis of M+4 enrichment in citrate, succinate, fumarate, and malate, represented as atom percent excess (APE), in C2C12s, KP-6634s, and KPH2-7215s cultured for 5 hrs with U-^{13}C-glutamine. N = 3 with 2 technical replicates. Data represent mean ± s.e.m.

In addition, aspartate may be elevated despite no significant differences in TCA cycle metabolites in sarcoma cells because of increased glutamate-oxaloacetate transaminase 2 (GOT2) expression. GOT1/2 act to generate aspartate from the TCA cycle (**see Supplementary Fig. 2E, 2G; p. 8, first paragraph**).

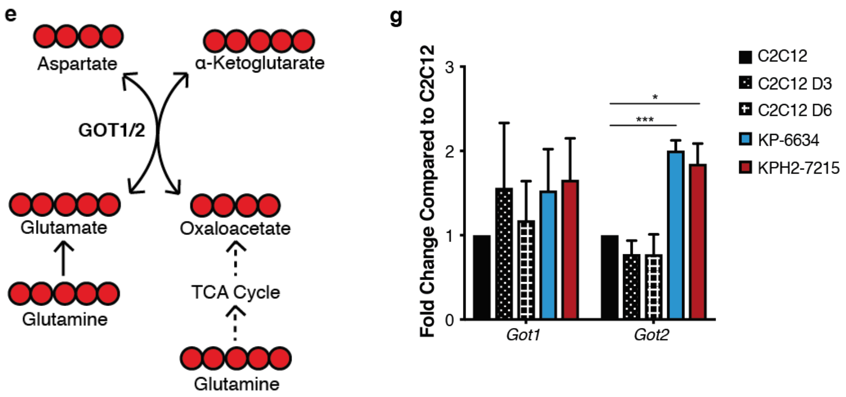

Supplementary Fig. 2: (E) Schematic depicting the transamination reaction generating aspartate by glutamate-oxaloacetate transaminase 1/2 (GOT1/2). Filled red circles represent ^{13}C atoms derived from U-^{13}C-glutamine.
(G) *Got1* and *Got2* mRNA levels in C2C12, C2C12 D3, C2C12 D6, KP-6634, and KPH2-7215 cells. N = 3 with 3 technical replicates. Data represent mean ± s.e.m. *p<0.05, ***p<0.0005.

3. The methods note that labeling is done with DMEM (and growth curves with 'media based on DMEM'), but DMEM traditionally contains large amounts of glutamine. Could the authors clarify whether these experiments were done in glucose/glutamine-free DMEM supplemented with labeled/unlabeled versions?

For labelling studies, glucose/glutamine-free DMEM supplemented with unlabeled glucose and labeled glutamine was used. For proliferation and viability assays, glucose/glutamine-free DMEM supplemented with the described metabolites was used. This has now been clarified in the relevant **Methods** sections (**see p. 17 – 18**).

4. The authors show levels of certain metabolites for some tumors and not others. I appreciate that not all data will be equivalently robust across distinct experiments, but could the authors provide a supplementary table with all of the acquired metabolomics data in which they are confident? This paper represents an important resource, and such a table will be of interest to readers in the field

These data are now provided as tables in **Supplementary Data 1** and **3**.

Reviewer #2 (Remarks to the Author); expert in sarcoma:

1A. The authors use GLS levels (mRNA and protein), to predict which sarcoma sub-types would, in principal be sensitive to GLS inhibition. However, are GLS levels a good predictor of CB-839 sensitivity? Although it is reasonable to target GLS in GLS-high tumors, it does not appear to be a consensus in the literature for biomarkers that could predict sensitivity to CB-839. See for example (PMID: 31088535), in which CB-839 resistant breast cancer lines and xenografts exhibit high GLS mRNA and protein levels. This caveat should be discussed.

The reviewer is correct in that a number of studies where GLS is expressed, these tumours are not sensitive to GLS inhibition. A possible scenario for this insensitivity is that they use glycolysis instead of glutaminolysis for anaplerotic feeding of the TCA cycle. This would allow cells to overcome GLS inhibition to meet their metabolic/biosynthetic needs. However, GLS inhibition does not capture the full scale of glutamine metabolism in cancer and the response to such inhibition remains highly dependent on the tumour microenvironment and genetic background of these tumours.
This has now been expanded in our discussion (**see p. 14, second paragraph**).

1B. Additionally, to say in the abstract that "Multiple STS subtypes expressing elevated glutaminase (GLS) levels are highly sensitive to glutamine starvation" does not fully reflect what was tested. The same laboratory has previously used another human liposarcoma cell line in their studies (LPS246, is this line one resistant too?), and at least one more leiomyosarcoma cell line available at ATCC. Those, and if possible additional ones, should be tested for glutamine starvation and CB-839 treatment in vitro (alongside with GLS protein levels). If there is

variability across the few lines available, the authors should simply acknowledge that more studies in additional sarcoma models will be required to more comprehensively understand these differences.

We further tested *in vitro* the effects of glutamine deprivation and CB-839 treatment on CCL-136 (rhabdomyosarcoma), SK-UT-1B (leiomyosarcoma), LPS246 (dedifferentiated liposarcoma), and T778 (well-differentiated liposarcoma) human cell lines.

Much like HT1080s and SK-LMS-1s, a decrease in proliferation and viability was observed upon glutamine deprivation in SK-UT-1, and CCL-136 cells. Similar to SW872s, only glucose withdrawal affected LPS246 cell proliferation, while viability was unaffected upon glutamine withdrawal. In contrast to the other liposarcoma cell lines, glutamine deprivation decreased T778 proliferation, however, no effect was observed on cell viability, suggesting this results in growth arrest rather than cell death. (**see Supplementary Fig. 1I – J; p. 6 – 7, third paragraph**). Consistent with glutamine deficiency, SK-UT-1B and CCL-136 proliferation and viability were decreased upon CB-839 treatment, while T778s showed lowered cell proliferation with no changes to cell viability. In addition, CB-839 did not significantly impact LPS246 cell proliferation or viability (**see Supplementary Fig. 5A – B; p. 10, second paragraph**). Altogether, these results demonstrate that glutamine deprivation inhibits the *in vitro* growth and viability of multiple STS cell types and not all sub-types behave in a similar manner

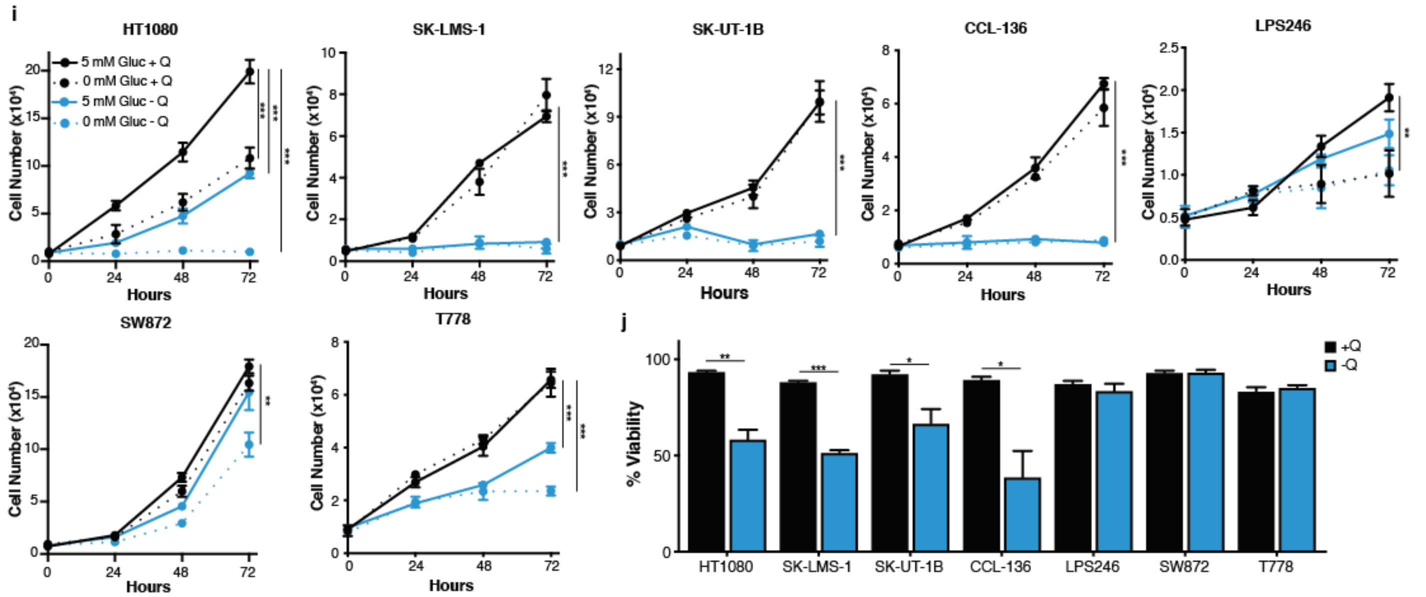

Supplementary Fig. 1: (I) Proliferation of human HT1080s, SK-LMS-1s, SK-UT-1Bs, CCL-136s, LPS246s (top row, left to right), SW872s, and T778s (bottom row, left to right) grown in media with or without glucose (Gluc) and/or glutamine (Q). N = 3 with 3 technical replicates. Data represent mean ± s.e.m. **p<0.005, ***p<0.0005.
(J) Viability of HT1080s, SK-LMS-1s, SK-UT-1Bs, CCL-136s, LPS246s, SW872s, and T778s grown in media with or without Q and assessed after 48 hrs. N = 3 with 2 technical replicates. Data represent mean ± s.e.m. *p<0.05, **p<0.005. ***p<0.0005.

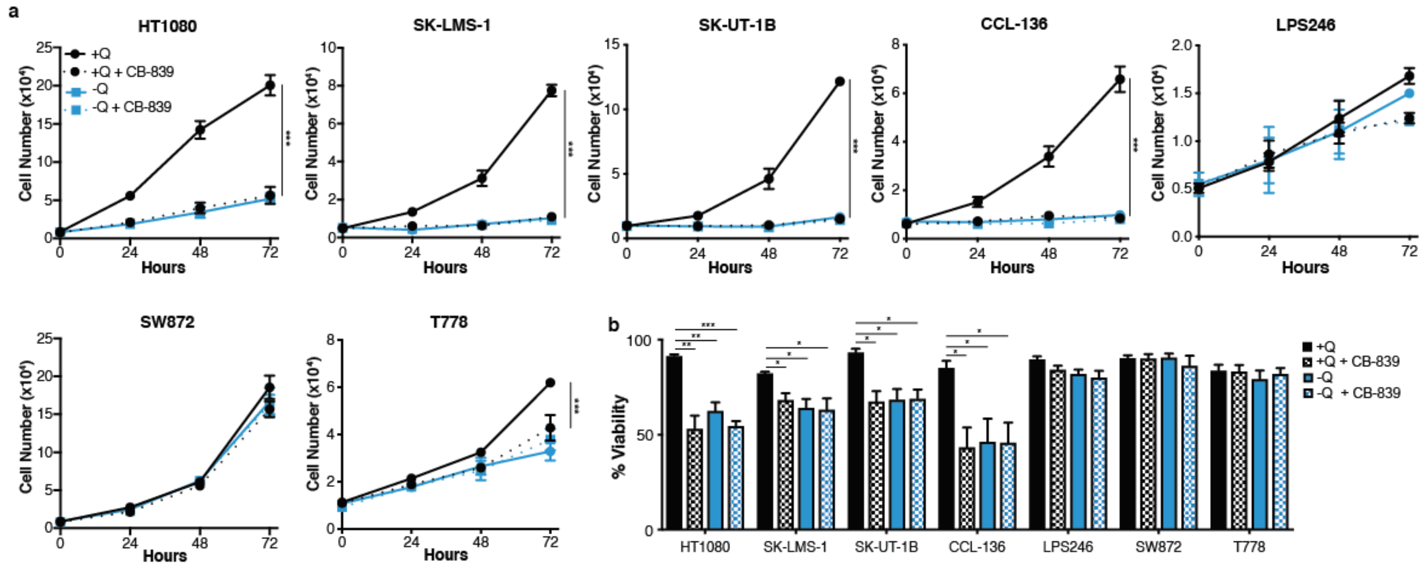

Supplementary Fig. 5: (A) Proliferation of HT1080s, SK-LMS-1s, SK-UT-1Bs, CCL-136s, LPS246s (top row, left to right), SW872s, and T778s (bottom row, left to right) grown in media with or without glutamine (Q) and treated with 1 μM CB-839. N = 3 with 3 technical replicates. Data represent mean ± s.e.m. ***p<0.0005.
(B) Viability of HT1080s, SK-LMS-1s, SK-UT-1Bs, CCL-136s, LPS246s, SW872s, and T778s grown in media with or without Q, treated with 1 μM CB-839 and assessed after 48 hrs. N = 3 with 2 technical replicates. Data represent mean ± s.e.m. *p<0.05, **p< 0.005, ***p<0.0005.

GLS levels in these cell lines were also assessed by Western blot (**see Supplementary Fig. 4D; p. 9, second paragraph**). In line with the proliferation assays, the newly included SK-UT-1Bs and CCL-136s (sensitive lines) express high GLS levels. Interestingly, much like the differential responses observed in the proliferation assays, liposarcoma lines showed varied GLS expression with LPS246 and T778 having GLS, despite only T778 being partly sensitive to glutamine deprivation or CB-839 treatment.

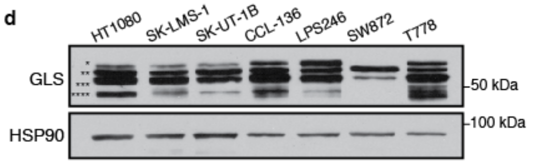

Supplementary Fig. 4: (D) GLS protein abundance in HT1080s, SK-LMS-1s, SK-UT-1Bs, CCL-136s, LPS246s, SW872s, and T778s. *, KGA isoform; **, unspecific band; ***, GAC isoform; ****, unspecific band; as determined by manufacturer's datasheet.

These differential responses to glutamine deprivation and CB-839 in the liposarcoma cells, may be dependent on the liposarcoma type they are derived from (LPS246, dedifferentiated liposarcoma; SW872, pleomorphic liposarcoma; T778, well-differentiated liposarcoma), in addition to other metabolic adaptations that differentiate them from the fibrosarcoma, leiomyosarcoma, and rhabdomyosarcoma cell lines.

2. Would it be possible that sarcomas with RAS mutations show greater dependency on glutamine metabolism (which could also reflect different GLS levels across sub-types)? Do the 2 sensitive human cell lines harbor Ras mutations? There are other sarcoma datasets available that provide both mutational analysis and mRNA expression analysis (TCGA sarcoma data set).

Based on information available through CCLE (The Broad Institute of MIT & Harvard) and ATCC, of the sensitive lines, HT1080 and CCL-136 harbour an *NRAS* mutation, while SK-LMS-1 and SK-UT-1B do not possess any Ras mutations. LPS246 show *MDM2* amplification, while T778 demonstrate *MDM2*, *CDK4*, and *HMGA2* amplification. Interestingly, the resistant SW872 liposarcoma line possesses a *BRAF* mutation, which would similarly activate the Ras signaling pathway. This suggests that dependency on glutamine metabolism is not completely reliant on Ras mutations.

Furthermore, based on TCGA analysis (Abeshouse et al., 2017), potentially functional mutations found in the surveyed sarcomas included truncating mutations in *NF1*, *NF2*, and *PRKDC*. In the same study, dedifferentiated liposarcomas were

defined by 12q13~15 amplifications, including highly recurrent copy-number gains or amplification of *MDM2*, *CDK4*, and *HMGA2*. For the case of leiomyosarcomas, deletions or mutations of *TP53*, *RB1*, and *PTEN* were found. Analysis in UPS samples found high-level amplification of *CCNE1*, *VGLL3*, and *YAP1*, as well as a YAP1/VGLL3 target gene signature.

Altogether, this indicates the responsive to CB-839 is not solely dependent on Ras mutations (genetic background), but rather the cell of origin and/or microenvironment. This has now been included in the discussion of the main text (**see p. 14, second paragraph**).

3. It would be of interest to performed long-term proliferation assays *in vitro* with CB-839 (longer than 72h) in the sarcoma lines to determine if they remain sensitive or are equally able to re-wire metabolic dependencies which could also inform about the requirement for combination therapies.

To address this, C2C12s, KP-6634s, KPH2-7215s, and HT1080s were cultured in media with or without CB-839 for 8 days (192 hours). Indeed, during this culture period, the sarcoma cells were unable to proliferate as in the short-term experiments. C2C12s again also showed slower proliferation rates (**see Supplementary Fig. 5C; p. 10, second paragraph**).

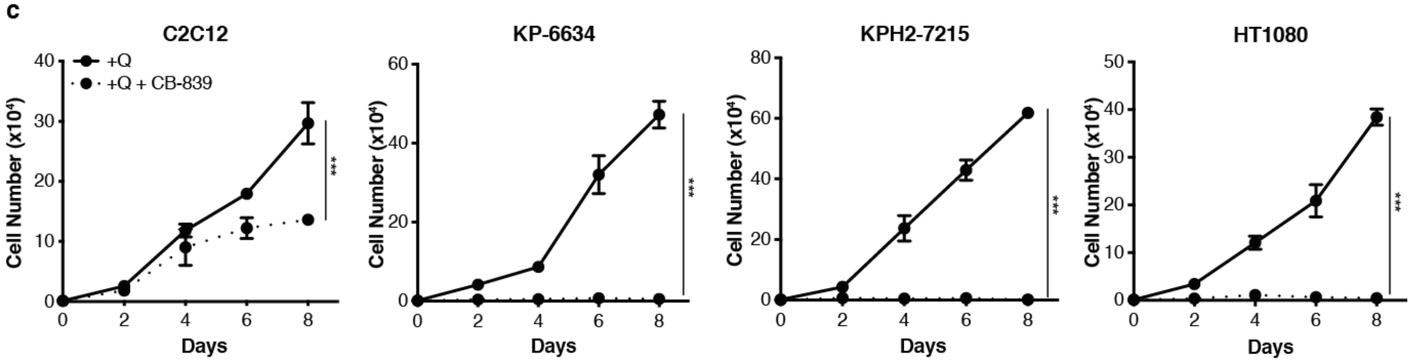

Supplementary Fig. 5: (C) Proliferation of C2C12s, KP-6634s, KPH2-7215s, and HT1080s (left to right) grown in media with Q and treated with 1 μM CB-839 for 8 days. N = 3 with 3 technical replicates. Data represent mean ± s.e.m. ***p<0.0005.

Minor points:

- The authors re-use images from their previous publication in Nature communications (reference 41) with Figure S1A and S1F here corresponding to Figure 1B and 1D in ref 41. While the authors clearly state this model was previously described and in the figure legends that the images were adapted from the previous publication, it seems unnecessary to re-include the same images. Furthermore, while the representative tumors images and the same between studies, the graphs depicting tumor weight suggest they correspond to different cohorts (please see Fig1B in this study and Fig 1B in ref. 41).

The images have now been removed from **Supplementary Fig. 1** to avoid confusion.

- Page 11: “However, in marked contrast to previous in vivo studies, CB-839 administration to KP and KPH2 animals substantially inhibited tumour growth, as calculated from the difference in the muscular compartment of tumor-bearing limbs (red) relative to control limbs (green) (Fig. 7A-C; 7D-F).” Which in vivo studies are the authors referring here? There is no reference for this statement and is unclear whether the authors are refereeing to studies using sarcoma lines or other types of tumors (eg. PDAC).

References to previous studies are now included for clarification (**see p. 12, first paragraph)**.

- How did the authors choose a dose of 2uM for CB-839 for all lines analyzed? Were IC50 values determined, or it was based on previous studies.

The dose used for the studies is 1 μM, based on previous studies (Gross et al., 2014; Jacque et al., 2015; Momcilovic et al., 2017). This has now been corrected in the Methods (**see p. 17**) and relevant Figure legends (**see p. 28**).

- In the discussion: "Although human STSs show significant heterogeneity of GLS expression (Supplementary Fig. 4D-E), we noted a striking enrichment in UPS (Fig. 4F-H)". There is currently no Figure 4H (both graphs are depicted as Figure 4F).

This has now been corrected in the discussion (**see p. 13, second paragraph**).

- In some human cancer tissues, increased levels of GLS1 are associated with a higher disease stage and poor prognosis PMID:25844758. Did the authors see a similar trend in samples analyzed in Fig 4E (if clinical data is available).

Clinical data (disease stage) for these samples are not available from Biomax. While survival data were not sufficiently available in the aforementioned datasets (**Fig. 4F; Supplementary Fig. 4H**), the dataset by Gibault *et al.* provided *GLS* expression for leiomyosarcoma patients with disease grade information. Poorly differentiated (PD) leiomyosarcoma showed lowest *GLS* levels, which increased with disease grade in well-differentiated (WD) patients (**see Fig. 4G; p. 10, first paragraph**).

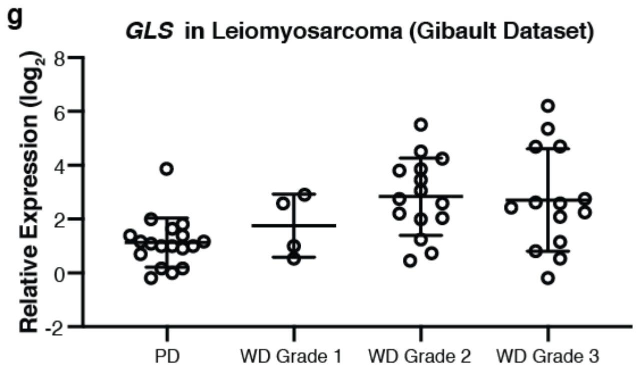

Fig. 4: (G) *GLS* mRNA expression from Oncomine analysis of Gibault *et al.* leiomyosarcoma patient samples datasets. Values are normalized to median-centered intensity and shown on a log_2 scale. PD, poorly differentiated; WD, well-differentiated.

REVIEWERS' COMMENTS:

Reviewer #1 (Remarks to the Author):

The revised manuscript addresses most of the reviewer comments and the inclusion of these revisions and the raw metabolomics data are appreciated. A few minor points remain:

1. Regarding the 13C-glutamine tracing to TCA cycle intermediates, the addition of additional TCA cycle intermediates is welcome. The authors note in the text that the fact that aspartate incorporates more glutamine-derived 13C than other TCA cycle intermediates could be the result of transamination. However, as currently written, this statement and Supplementary Fig. 2e might be misleading to a non-expert as it appears at first glance to support the notion that transamination from glutamate provides carbon, when it only provides nitrogen needed to form aspartate from oxaloacetate. It would be helpful for the schematic to emphasize that when tracing carbons, the only precursor for aspartate is oxaloacetate (derived either from oxidative glutamine metabolism via succinate/fumarate/malate or reductive glutamine metabolism via citrate/oxaloacetate). The text and figure should be modified to clarify this potential point of confusion. While it is surprising that aspartate is more labeled than its precursors, I agree that there are potential explanations and this does not have to be completely resolved.

2. A related, minor point: the authors comment that glucose-derived alanine production could explain why glutamine-derived nitrogen is incorporated into alanine at a higher fractional percentage compared to aspartate. If I'm reading the axes correctly, the data show that about 40% of aspartate is made de novo from glutamine-derived carbon in the course of the experiment, but only 4% or so from glutamine-derived nitrogen. Is there another nitrogen source for aspartate? The critical point of the authors stands—this incorporation is increased in sarcoma relative to normal cells—but overall glutamine appears to be a minor contributor to aspartate nitrogen. For both this and the above point, it will be helpful for the authors to include in the methods more details about the GC-MS tracing including natural isotope correction.

3. My apologies for missing this in the first round: the legends or methods should clarify how metabolite analyses are normalized.

4. There is a lengthy discussion about potential combinatorial therapeutic strategies that culminates with the conclusion that PARP inhibition might synergize with CB-839. Absent any data directly testing this hypothesis, the conclusions of this discussion should be modified to emphasize that support for this combinatorial therapy is purely speculative and that future studies should test this directly.

Reviewer #2 (Remarks to the Author):

The authors added the experiments suggested and have addresed the points raised in the resulst and discussion.

Reviewers' Comments:

Reviewer #1 (Remarks to the Author):

The revised manuscript addresses most of the reviewer comments and the inclusion of these revisions and the raw metabolomics data are appreciated. A few minor points remain:

1. Regarding the 13C-glutamine tracing to TCA cycle intermediates, the addition of additional TCA cycle intermediates is welcome. The authors note in the text that the fact that aspartate incorporates more glutamine-derived 13C than other TCA cycle intermediates could be the result of transamination. However, as currently written, this statement and Supplementary Fig. 2e might be misleading to a non-expert as it appears at first glance to support the notion that transamination from glutamate provides carbon, when it only provides nitrogen needed to form aspartate from oxaloacetate. It would be helpful for the schematic to emphasize that when tracing carbons, the only precursor for aspartate is oxaloacetate (derived either from oxidative glutamine metabolism via succinate/fumarate/malate or reductive glutamine metabolism via citrate/oxaloacetate). The text and figure should be modified to clarify this potential point of confusion. While it is surprising that aspartate is more labeled than its precursors, I agree that there are potential explanations and this does not have to be completely resolved.

We agree that the schematic (originally **Supplementary Fig. 2e**) is misleading with the surrounding data. Therefore, we have opted to modify the original **Fig. 2g** and move it to **Supplementary Fig. 2e** to emphasize the transfer of nitrogen in the transamination reaction. Readers can use the schematic in **Fig. 2c** to understand the movement of carbons from glutamine. We have rephrased the paragraphs to clearly state that glutamine-derived carbons into aspartate are through oxidative glutamine metabolism and not transamination (**see p. 7 – 8**).

2. A related, minor point: the authors comment that glucose-derived alanine production could explain why glutamine-derived nitrogen is incorporated into alanine at a higher fractional percentage compared to aspartate. If I'm reading the axes correctly, the data show that about 40% of aspartate is made de novo from glutamine-derived carbon in the course of the experiment, but only 4% or so from glutamine-derived nitrogen. Is there another nitrogen source for aspartate? The critical point of the authors stands—this incorporation is increased in sarcoma relative to normal cells—but overall glutamine appears to be a minor contributor to aspartate nitrogen. For both this and the above point, it will be helpful for the authors to include in the methods more details about the GC-MS tracing including natural isotope correction.

Based on the formulation provided by ThermoFisher Scientific, the base media used for the labelling studies still contains branched chain amino acids (leucine, isoleucine, valine) as well as other amino acids such as serine and glycine, which could be a nitrogen source for aspartate. We have now included this into the main article (**see p. 8, second paragraph**) and more detailed methods about the GC-MS tracing and natural isotope correction in the **Stable Isotope Metabolite Tracing** paragraph of the **Methods** section (**see p. 19**).

3. My apologies for missing this in the first round: the legends or methods should clarify how metabolite analyses are normalized.

The **Metabolomic Analysis** paragraph in the **Methods** section has been expanded to include details on how the metabolic analyses were normalized (**see p. 19 – 20**).

4. There is a lengthy discussion about potential combinatorial therapeutic strategies that culminates with the conclusion that PARP inhibition might synergize with CB-839. Absent any data directly testing this hypothesis, the conclusions of this discussion should be modified to emphasize that support for this combinatorial therapy is purely speculative and that future studies should test this directly.

We have modified the concluding paragraph of the Discussion to emphasize that the combination of CB-839 with PARP inhibition are currently purely speculative and future studies are required (**see p. 15, second paragraph**).
